# Emerging Technologies for Investigating Food Consumer Behavior: A Systematic Review

**DOI:** 10.1111/1541-4337.70340

**Published:** 2025-11-16

**Authors:** Kyriaki Kechri, Christina Kleisiari, Leonidas Sotirios Kyrgiakos, Marios Vasileiou, Dimitra Despoina Tosiliani, Vasileios Angelopoulos, George Kleftodimos, George Vlontzos

**Affiliations:** ^1^ Department of Agriculture, Crop Production and Rural Environment University of Thessaly Volos Greece; ^2^ Mediterranean Agronomic Institute of Montpellier (CIHEAM‐IAMM) Montpellier France

**Keywords:** big data, consumer insights, digital analytics, food sector, Virtual Reality

## Abstract

The evolving nature of food preferences and consumption patterns highlights the need for ongoing research in food consumer behavior. Most existing research relies on traditional methods, like questionnaires, which are often costly, time‐consuming, and prone to bias. The increasing integration of emerging technologies, including Artificial Intelligence (AI), Virtual Reality (VR), Social Media Analytics (SMA), and Big Data, into the food sector presents novel opportunities to overcome these limitations. This systematic review, conducted in accordance with the Preferred Reporting Items for Systematic Reviews and Meta‐Analyses (PRISMA) guidelines, identified 628 records, out of which 159 eligible articles were included. These examine how technological innovations contribute to understanding food trends and consumption preferences, as well as consumer attitude towards these technologies. The findings reveal the considerable potential of big data, SMA, and transactional analytics to provide large‐scale, diverse, and real‐time data into consumer behavior. Machine Learning (ML) techniques improve the analysis and interpretation of such complex datasets, enabling high predictive capability and a more precise market segmentation to provide consumers with personalized marketing content. Immersive technologies like VR offer realistic simulations of food purchasing behaviors and adapt to multiple research scenarios, overcoming limitations of traditional research methods. However, most technology‐based studies remain primarily quantitative, limiting depth of understanding. Challenges in automated data interpretation, reduced sensory immersion in VR environments, users’ unfamiliarity and data privacy concerns need also to be addressed. Thus, future research should focus on technological advancement, improving usability and establishing ethical frameworks to foster consumer trust, while also integrating qualitative methods too beyond only relying on technology‐based outcomes.

**Abbreviations**
AI: Artificial Intelligence
VR: Virtual Reality
SMA: Social Media Analytics
PRISMA: Preferred Reporting Items for Systematic Reviews and Meta‐Analyses
ML: Machine Learning
IoT: Internet of Things
RQ: research question
SLR: systematic literature review
WoS: Web of Science
R&D: research and development
TL: transfer learning
UCG: user‐generated content
NLP: Natural Language Processing
EEG: electroencephalography
fMRI: functional magnetic resonance imaging
WTP: willingness to pay
TAM: Technology Acceptance Model
TPB: theory of planned behavior
PCT: privacy calculus theory
SOR: Stimulus‐Organism‐Response
SERVQUAL: service quality model
SentiStrength: sentiment strength detection
CFA: confirmatory factor analysis
EFA: exploratory factor analysis
PCA: principal component analysis
SEM: structural equation model
TF‐IDF: Term Frequency—Inverse Document Frequency
SVM: Support Vector Machine
XGBoost: Extreme Gradient Boosting
DBSCAN: density‐based spatial clustering of applications with noise
LDA: Latent Dirichlet Allocation
PIC: photo‐enhanced imaginative condition

## 1 Introduction

Understanding food‐related consumer behavior is essential for retailers to meet consumer needs, design successful marketing strategies, and remain competitive (Oszust and Stecko 2020; Johnson 2024). However, most research on consumer behavior includes questionnaire‐based surveys, which are time‐consuming and often limited by participant recruitment challenges and selection bias. Technology‐based approaches offer promising solutions to address these issues. Supporting information

In recent years, many innovations have been integrated in the food sector, such as Internet of Things (IoT), AI, ML, Robotics, Augmented Reality, VR, and Big Data Analytics, providing both retailers with valuable consumer insights and consumers with enhanced services (Krishen et al. 2021; Ma et al. 2024; Stecuła et al. 2024; Tao et al. 2020; Yakut 2022). In 2023, food retailers invested on average 0.9% of their total sales on technology (FMI 2024). AI investment reached $11.08 billion in 2024 in the food and beverage industry and is projected to rise to $236.8 billion in the next decade (Precedence Research 2025), with most food retailers using AI for customer data collection and analysis, pricing decisions, and personalized marketing (FMI 2024). Notably, personalization appears among consumer trends of 2025 (Statista 2025). Immersive technologies are expanding too, with global VR and AR investments reaching $47.58 billion in 2024, which amount is also expected to increase (Precedence Research 2024).

This growing technological interest, along with the need for ongoing research on food consumption behavior driven by the evolving nature of consumer patterns, and the limitations of traditional research methods, makes it essential to explore the potential of new technologies for investigating food consumer behavior. In this context, Tao et al. (2020) demonstrated how big data analytics, mainly through advanced text mining techniques, contribute to food‐related consumer‐opinion mining, new food product development and recognition of dietary habits, using digital sources like social media. Moreover, the review of Bhavadharini et al. (2023) is a critical reference, indicating how immersive technologies can be used for uncovering consumer responses to food‐related stimuli through controlled and realistic simulations of consumer behavior. Lastly, Rantala et al. (2022) highlighted the great potential of wearable and sensory‐based applications in objectively identifying food‐evoked emotions and eating choices, minimizing biases of self‐reported data.

Although these reviews offer valuable insights, they mostly tend to specialize on certain technological types or narrow aspects of consumer behavior, such as emotional responses. In contrast, this review adopts a more holistic approach, examining and comparing the effectiveness of a broader range of technologies, like big data, transactional analytics, personalized systems, VR, ML, SMA, in capturing multiple behavioral parameters, such as consumers’ health and environmental awareness in the context of food consumption, as well as perceptions on various food product attributes, like price, food quality, and taste. Furthermore, while several case studies compared the effectiveness of technologies like VR in capturing consumer behavior to traditional approaches, like questionnaire‐based surveys (Fang et al. 2021; Lombart et al. 2020; B. Wang et al. 2023), this comparison has not been conducted at a systematic level through a literature review. This review aims to fill these research gaps, mainly focusing on addressing the following research questions (RQs):

1. Which behavioral dimensions of food consumption can be captured via emerging technologies and how can the collected data be analyzed and interpreted?
2. What are the key benefits and challenges of using these technologies in food‐related consumer research compared to traditional research methods?
3. Based on these challenges and methodological gaps, what are the future research directions?
4. How do consumers perceive the integration of new technologies enabling consumer behavior analysis in the food sector?

## 2 Methodology

To answer the above‐mentioned RQs, a systematic literature review (SLR) was conducted to ensure the precise assessment of existing literature, uncover research gaps, and provide future research guidelines (Snyder 2019). This SLR follows the PRISMA guidelines as a methodological tool (Page et al. 2021).

### 2.1 Data Sources

Scopus and Web of Science (WoS) databases have been selected as data sources for this review due to their large coverage of literature about fields like marketing, consumer behavior, food science, and technology. Their journal selection criteria ensure the inclusion of high‐quality articles, while the advanced search‐functions offered in these databases enable the precise identification of relevant studies (Pranckutė 2021).

### 2.2 Search Strategy

The search strategy of this SLR was designed to integrate three main dimensions, particularly: a) consumer behavior, b) the food sector, and c) emerging technologies that support consumer behavior tracking and analysis. Keywords indicative of consumer behavior parameters, such as “consumer behavior,” “consumer behavior” (American and English form of the same word), “consumer perceptions,” “consumer preferences,” and “consumer attitude,” were selected. These were combined with keywords related to food consumption, including “food,” “nutrition,” “eating,” “grocery,”, “groceries,” among others, and with keywords indicative of modern technologies used in consumer behavior analysis to reflect the technological dimension. More precisely, a broad set of technological terms, including “Artificial Intelligence,” “Virtual Reality,” “Internet of Things,” and “Big Data,” have been integrated in the query string both as the full terms and abbreviations (AI, VR, and IoT) to explore the benefits and challenges of analyzing consumer behavior through these means.

For the optimization and precision of the search results, Boolean operators (AND, OR) have been used within the databases to include and combine the selected search terms (Sampson et al. 2009). Titles, abstracts, or keywords were the primary sections where the search terms needed to appear, ensuring in this way the identification of the most relevant articles for the research objectives.

### 2.3 Inclusion and Exclusion Criteria

Applying this search strategy revealed negligible research in the field until the early 2010s, with an exponential growth of relevant publications starting in 2014. Thus, only publications after 2014 were included in the review. The selection was further limited to journal articles, written in English, leading to the identification of 628 articles in total—343 from Scopus and 285 from WoS.

For easier data handling, R studio has been used. However, during data import, three articles from Scopus could not be processed, reducing the number to 625. After the automatic removal of 186 duplicated documents in R Studio, the two datasets have been merged into one single, leaving 439 unique articles for screening.

Title and abstract screening, as well as full text assessment, were based on predefined inclusion and exclusion criteria. Specifically, studies were included if they:

i. Assessed food consumer behavior through technological means (e.g. VR‐based experiments, or SMA), thereby addressing one or more of RQs 1–3, or
ii. Examined consumer perceptions of using such technologies (e.g. surveys on consumer willingness to use personalized nutrition applications that simultaneously collect users’ dietary data), thus addressing mainly the 4th RQ.

In contrast, studies not addressing any of the RQs, such as those simply mentioning the selected search terms in unrelated contexts (e.g. the application of emerging technologies in agricultural processes), as well as reviews, were excluded.

To ensure robustness, a double screening procedure was applied based on these predefined criteria, where two researchers independently assessed all articles at both PRISMA stages (screening and eligibility phase). An inter‐rater reliability check was performed, yielding a Cohen's kappa of 0.83. This indicates almost perfect agreement among researchers (Landis and Koch 1977). Any disagreements were resolved through discussion until consensus was reached.

As a result, at the title and abstract screening phase, 191 articles were excluded because they were out of scope. During the eligibility phase, a full‐text assessment of the 248 remaining articles was performed, resulting the exclusion of 89 articles, because:

- 67 had only a minor contribution to the research objectives,
- 17 were reviews, and
- full‐text access was unavailable for 5 articles.

**FIGURE 1:**
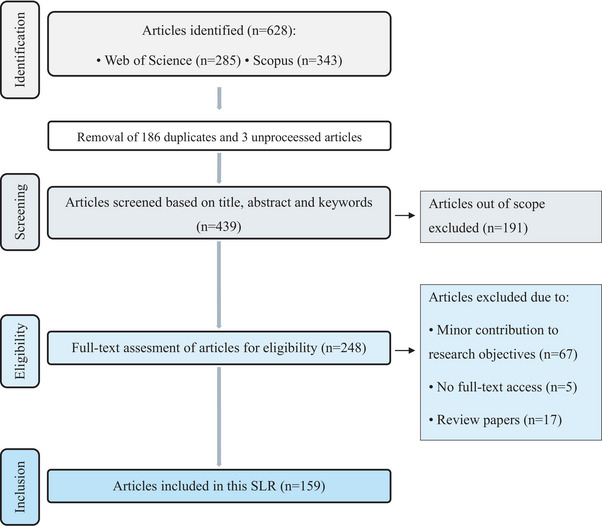
Systematic review methodology under PRISMA guidelines.

Finally, 159 articles met the eligibility criteria and were including in this SLR (Figure 1).

### 2.4 Data Extraction

**FIGURE 2:**
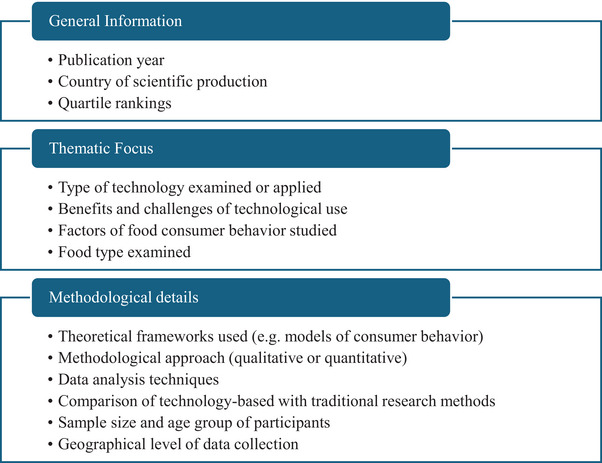
Overview of the variables assessed in the 159 reviewed articles.

Based on the RQs, as well as on the thematic categories and informational fields identified during the review of the 159 included studies, the variables examined in each article were organized into three main categories, as depicted in Figure 2. First, general information of the articles, including publication year, country of scientific production, and journal quartile, were systematically recorded. Second, topical information, particularly the type of technology addressed, associated benefits and challenges in capturing food‐related behavioral parameters and based on consumers’ perceptions, and food type examined, were also documented for each study to guide future research. Third, methodological information was captured in each study, including theoretical frameworks used in consumer research, sample, and age group of participants, geographical level of data collection, methodological approach (quantitative or qualitative), data analysis techniques employed, and whether technology‐based methods were compared with traditional research methods (S1).

The evaluation of these variables in each paper was independently performed by two researchers to ensure reliability, with any discrepancies being resolved through in‐depth discussion until consensus. This process facilitated a consistent and comparative synthesis across the literature, contributing to the study's objectives, by understanding how modern technologies can support the analysis of food‐related consumer behavior, and identifying consumer attitudes towards integrating such technologies in the food sector. Based on these findings, several research and methodological gaps have been identified, leading to recommendations for future research.

## 3 Results and Discussion

### 3.1 General Information of the Articles

#### 3.1.1 Annual Scientific Production

**FIGURE 3:**
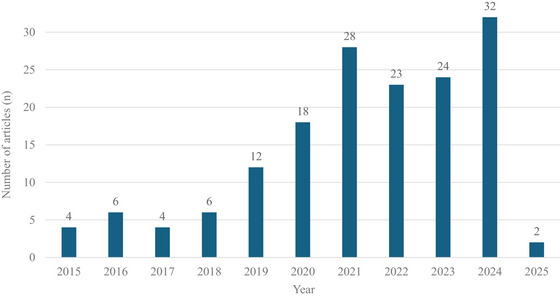
Distribution of the reviewed studies per publication year.

Figure 3 shows a growing research output on food consumer behavior and emerging technologies, with an average annual growth rate of 26% up to 2024. A particularly noticeable increase in relevant publications was observed in 2021, possibly reflecting the impact of Covid‐19 on technology adoption. Food retail chains rapidly adopted digital solutions to reduce physical interaction and adapt to the new consumer demands, which resulted in an increasing research interest in technological applications during this period. Social distancing measures may have also encouraged researchers to explore food consumer behavior through technology‐based approaches, such as big data and VR, as alternatives to direct interaction with consumers considering typical research and publication timelines, it is likely that such studies initiated in 2019 or 2020 were published in 2021, explaining the surge in that year (28 articles).

After this surge, the number of publications in 2022 and 2023 was quite lower and almost identical (23 and 24 articles, respectively), indicating a short‐term phase of stability. Such fluctuations are a common feature of research dynamics, as different growth periods are typically observed in the development of science (Larsen and von Ins 2010), with peaks and subsequent stagnation often influenced by major social or technological events and frequently followed by renewed growth (Bornmann et al. 2021). This renewed growth was observed in 2024 (32 articles), most probably due to the rapid evolution and integration of AI and related technologies into many aspects of everyday life.

#### 3.1.2 Scientific Production Per Country

**FIGURE 4:**
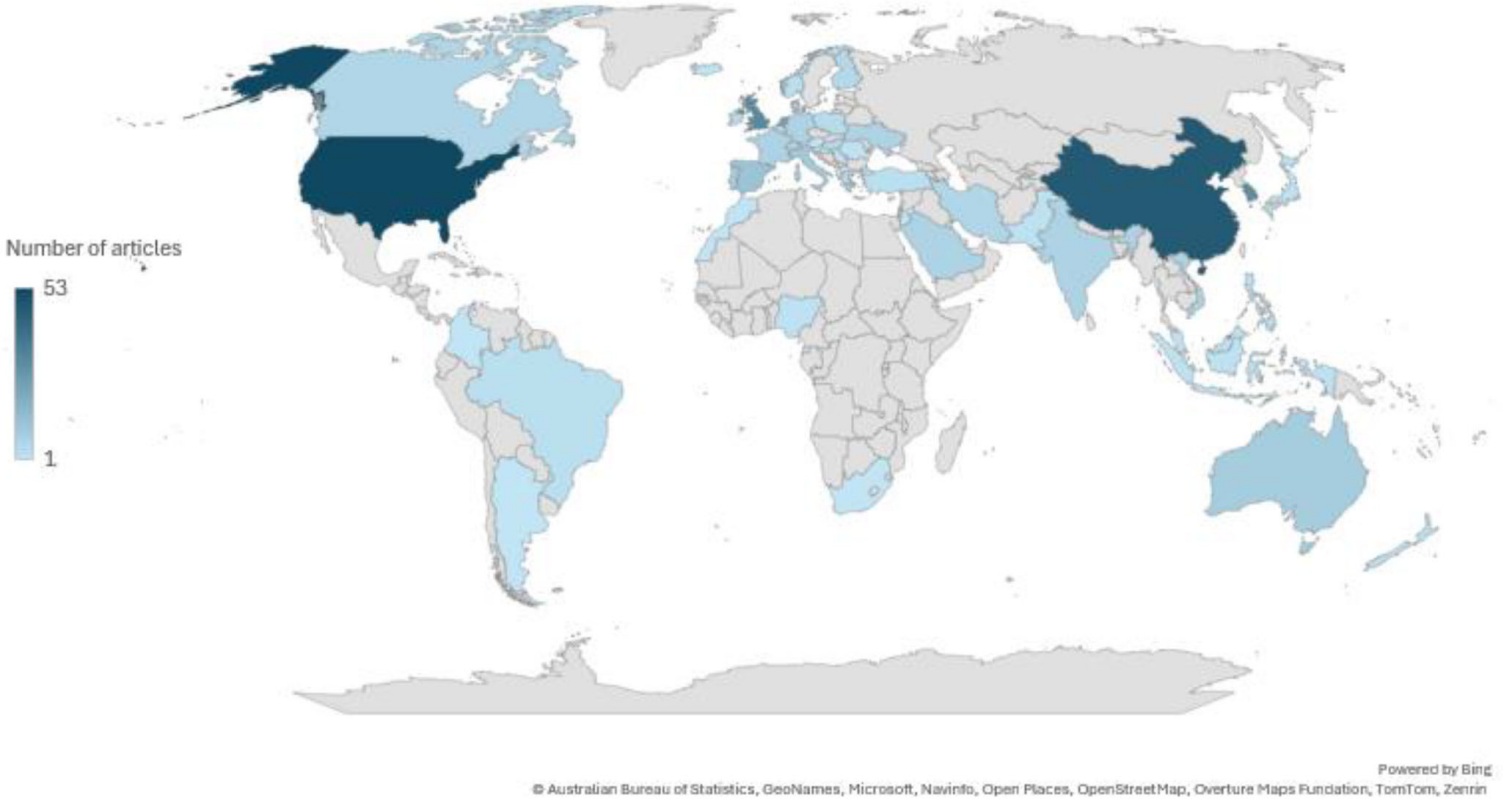
Scientific production per country in the reviewed articles. Darker shades represent higher numbers of articles, while lighter shades represent lower scientific production in the field of food consumer behavior and technologies.

**FIGURE 5:**
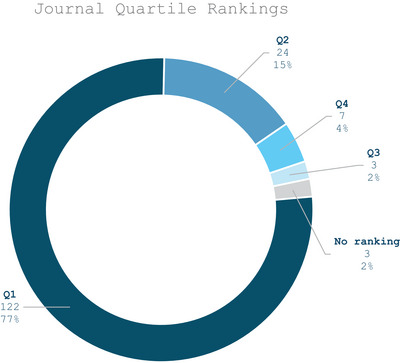
Quartile distribution of the 159 publications included in the review.

As illustrated in Figure 4, global scientific production in the field of food consumer behavior and technologies is mainly concentrated in the USA, China, South Korea, and the UK. This prevalence reflects their strong investment in research and development (R&D), with the USA and China together accounted for more than half of global R&D expenditures in 2022 (30% and 27%, respectively), and South Korea and the UK also ranking among the eight leading R&D performing countries worldwide (NSB 2025). Europe's contribution remains comparatively moderate, with the Netherlands standing out due to its strong public–private research linkages and international scientific collaborations (European Commission 2024). Research interest in innovative technologies is further reinforced by modern Dutch consumers’ demand for modern digital solutions, including online shopping, transparency tools, and AI‐based personalized applications (Capgemini Research Institute 2025). In contrast, scientific production in this area is limited for large parts of the world (Figure 5), exposing deep inequalities in scientific knowledge production globally.

#### 3.1.3 Quartile Rankings

The quality of the publications included in this review was assessed by examining the quartile rankings of the journals in which the 159 selected articles appeared. As depicted in Figure 5, the majority of studies (77%) were published in Q1 journals, followed by Q2 (15%). Only a small number of articles appeared in Q3 (2%) and Q4 (4%), while 2% were published in journals without a quartile classification. This distribution indicates that research on food consumer behavior and technologies is mainly concentrated in higher‐ranked journals, reflecting both the academic relevance of this topic and the visibility of the field in well‐established journals.

### 3.2 Topical Information of the Articles

#### 3.2.1 Technologies for Food‐Related Consumer Behavior Research

**FIGURE 6:**
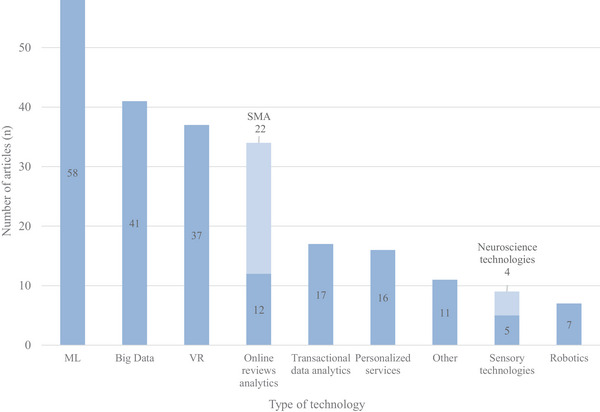
Emerging technologies enabling food‐related consumer behavior research: Distribution of the reviewed articles by type of technology applied or examined (ML = Machine Learning; VR = Virtual Reality; SMA = Social Media Analytics; "Other" includes technologies such as AI‐based facial recognition, augmented reality, video recording etc.).

The reviewed studies indicate a broad application of technological approaches in food‐related consumer behavior research, ranking from data‐driven technologies to immersive and sensor‐based tools (Figure 6). In this context, AI applications, particularly ML, stands out, appearing in about one third of the articles (≈35%) for enhancing data analysis and interpretation (Kanavos et al. 2018; Taghikhah et al. 2021; Nilashi et al. 2021; Hakim et al. 2021). In several cases, transfer learning (TL) was also performed, leveraging models trained on broader consumer datasets to optimize analytical processes and improve prediction in food‐related consumer behavior (Y. Chen and Zhang 2022; Chiu and Chuang 2021). The second most frequently used approach was Big Data analytics, appearing in 41 of the 159 included studies and often combined with SMA to extract insights from large‐scale consumer datasets (Mishra and Singh 2018; Singh and Glińska‐Neweś 2022; Y. Chen and Zhang 2022). The strong presence of both ML and Big Data highlights the increasing significance of automation in consumer research.

The third most frequently used technology was VR, applied in 37 studies to simulate realistic purchasing environments (Fang et al. 2021; Han et al. 2020; Lombart et al. 2019, Lombart et al. 2020; Siegrist et al. 2019; Zulkarnain et al. 2024). Online reviews analytics appeared in around one‐fifth of the studies (34 articles), with 22 extracting data from social media, mainly using Twitter as a source (Brandtner et al. 2021; S. Chen et al. 2023; Mishra and Singh 2018; Nilashi et al. 2021).

In 17 articles, transactional data from food retail stores were analyzed to identify objective purchasing behaviors (Vepsäläinen et al. 2022; Alawadh and Barnawi 2024), while 16 articles focused on personalized services, reflecting their growing significance in consumer analytics, but also consumer concerns around privacy (Kang and Namkung 2019; Peng et al. 2016; Su, Nguyen, et al. 2022; Geng et al. 2023). Sensory technologies have been effectively applied in a smaller number of articles to capture emotional responses to food‐related stimuli (van der Laan et al. 2022; Hakim et al. 2021). Lastly, 7 articles examined the integration of Robotics in the food retail, focusing on consumers’ perceptions of service‐bots (Chiu and Chuang 2021; Santiago et al. 2024).

##### 3.2.1.1 Data‐Driven Technologies (Big Data, SMA, Transactional Analytics, ML)

###### 3.2.1.1.1 Opportunities

The prevalence of big data and technologies, such as transactional analytics and SMA, among the studies of this review represents a valuable alternative to traditional research methods, as these technologies enable the collection of large amounts of consumer data characterized by high variety and velocity (Nilashi et al. 2021; Vepsäläinen et al. 2022; Alawadh and Barnawi 2024; Mishra and Singh 2018). Unlike surveys based on interviews or questionnaires, which are typically time‐consuming and constrained by limited participant recruitment, user‐generated content (UGC), such as online ratings and textual reviews on food products, services, and retail experiences, is abundant, globally available, and often freely accessible, allowing large‐scale and even cross‐national studies at relatively low cost (Chen et al. 2016; Culotta and Cutler 2016; Brandtner et al. 2021; X. Li et al. 2023). For instance, Jo and Lee (2021) used big data analytics to examine food values in Korea, China, and Japan, showing how large‐scale digital data can uncover both shared priorities, such as safety, and cultural differences that traditional surveys might miss.

Besides scale, data collected through digital platforms, mainly social media, are typically updated in real‐time, providing insights into shifting food preferences and market trends. In contrast, data from traditional research methods often become quickly outdated due to the rapidly changing nature of consumer attitudes (Demydyuk et al. 2024; Izang et al. 2019; Mishra and Singh 2018).

Equally important is the authenticity of data collected through these sources by capturing actual behaviors in natural settings. Social media platforms allow users to spontaneously express opinions on food products and services, reducing the risk of interviewer influence or participants’ reluctance to speak openly. Similarly, transactional records provide objective insights on actual spending patterns, rather than solely relying on self‐reported consumer responses, capturing discrepancies such as those between the reported willingness to pay (WTP) and real expenditures (Culotta and Cutler 2016; Brzustewicz and Singh 2021; Brandtner et al. 2021; Alawadh and Barnawi 2024).

In parallel, the increasing application of ML algorithms and other advanced techniques, such as Natural Language Processing (NLP), has transformed the analytical process, enabling researchers to automatically interpret large‐scale, complex, and often unstructured data, which would be unmanageable through conventional statistical techniques. Such approaches accelerate research process, while also increasing accuracy by minimizing the risks of researchers’ subjective interpretation during manual coding (Nilashi et al. 2021; Ju 2024; Tan et al. 2025). In this way, ML techniques can precisely predict food preferences and segment the market by identifying distinct consumer groups from large‐scale datasets, as shown by Salamzadeh et al. (2022), who analyzed grocery app data with ML algorithms to segment users by product category preferences and predict purchasing intentions. Their results demonstrated a predictive accuracy of 95%.

###### 3.2.1.1.2 Challenges

Although data‐driven technologies, like SMA enable the collection of large‐scale and diverse information, the quality of these data is sometimes undermined due to potential misinformation. UGC collected to reveal consumer attitudes may in some cases be fake, intentionally misleading, or driven by competitive motives, rather than reflecting users’ honest opinions. In addition, social media users rarely share demographic and socioeconomic details publicly, leaving many SMA‐based studies without this crucial information. Even when some demographic information is accessible in digital platforms, it lacks representativeness, as social media users are mostly young, limiting the generalizability of findings (Culotta and Cutler 2016; He et al. 2018; Fisk et al. 2024; Ariyasriwatana and Quiroga 2016).

Another limitation that should be acknowledged relates to the limited depth in understanding the underlying drivers of food‐related behavioral outcomes when solely relying on digital processes. Mishra and Singh (2018) confirmed this in a case study that used Twitter analytics to explore causes of food waste in the beef supply chain. Although analyzing users’ negative reviews identified potential factors of food waste, the casual nature and character limitations of Twitter posts limited the depth of results, making it difficult to explain deeper drivers of food waste behaviors. Similarly, transactional records, while capturing food spending patterns with precision, their analysis cannot explain behavioral mechanisms behind purchasing patterns (Clark et al. 2021; Grummon and Taillie 2017).

Besides concerns about data quality and explanatory depth, automated data analysis and interpretation remain also challenging. Although ML algorithms can handle big and complex datasets, their value is constrained by the need for high computational resources and advanced technological expertness due to their complexity. Moreover, they significantly depend on the availability of large and high‐quality datasets. Poor and limited data may undermine their performance and research reliability, while also increasing the risk of overfitting. Models trained on limited or low‐quality datasets often fail to adapt and generalize to new data and research contexts (Taghikhah et al. 2021; Nilashi et al. 2021; Natsume and Okamoto 2024; Sukumaran and Majhi 2024). In addition, NLP techniques cannot accurately explain some complex linguistic expressions, particularly those including sarcasm, irony, or indirect language, increasing the risk of data misinterpretation (Brzustewicz and Singh 2021; Fisk et al. 2024).

##### 3.2.1.2 Personalized and Robotic Systems

###### 3.2.1.2.1 Opportunities

Personalized and robotic systems, while less common than data‐driven or VR‐based approaches in the reviewed articles, represent a valuable alternative to traditional research, focusing on how customization and automation can improve consumer experience, data collection, and business efficiency. Personalized systems, increasingly delivered by food platforms, offer access to consumers’ transaction history and real‐time location to automatically recommend preferred products, menu options, and tailored coupons at the right moment and in nearby stores. Such targeted advertisements help consumers access the information needed far more quickly without being interfered from irrelevant cues, thus improving navigation and perceived service quality (Kang and Namkung 2019; Su, Nguyen, et al. 2022).

At the same time, retailers also benefit from personalization, as these systems generate insights into consumer demographics and their links to purchasing patterns, derived from transaction and interaction data. This supports the identification of market trends, the segmentation of distinct consumer groups, the automated targeting of potential customers through customized advertising, and the design of new products aligned with consumer demand. Finally, personalization enhances customers’ sense of uniqueness and loyalty, strengthening their relationship with companies. Empirical studies showed that this effected is mediated by trust, which drives continuance in using personalized food applications (Kang and Namkung 2019; M. T. T. Nguyen, Emberger‐Klein, et al. 2021).

Besides the improved marketing framework, personalized nutrition applications significantly improve dietary patterns. After users input data in such applications, such as eating habits, food preferences, and health‐related goals, these tools provide them personalized nutritional guidelines, aligned with their individual characteristics and needs. At the same time, they enable researchers to generate valuable insights into emerging eating patterns and consumer behavior (Peng et al. 2016; Berezowska et al. 2015; Pérez‐Troncoso et al. 2021; Reinders et al. 2020).

Personalized systems are often integrated into robotic technologies too. Self‐service technologies, such as AI‐based kiosks, offer faster, customized, and contactless services, while also stimulating users’ interest as novel tools. Although mainly used to improve convenience and enhance consumer satisfaction, these technologies also support consumer research by automatically storing customer data and generating insights that help retailers understand consumer behavior (Rasheed et al. 2023; Song and Kim 2022; Sung and Jeon 2020; Chiu and Chuang 2021).

###### 3.2.1.2.2 Challenges

Despite these benefits, consumers remain hesitant toward robotics and personalized services. Many are still unaccustomed to interacting with service‐bots, partly due to their preference for human interaction in food retail and concerns about robots’ ability to understand needs and solve complex problems (Sung and Jeon 2020; Song and Kim 2022; Rasheed et al. 2023). This unfamiliarity is further closely linked with technological anxiety, especially in online transactions or other services requiring users to share personal data, raising concerns about security and privacy (Habib and Hamadneh 2021; Berezowska et al. 2015). There is a part of them who even hesitate to register in food and nutrition apps due to the need to provide some personal information during registration (Peng et al. 2016; Berezowska et al. 2015; Reinders et al. 2020). Similar trust issues also appear in interactions with self‐service AI‐driven bots, which automatically store data on individual food preferences (Song and Kim 2022; Rasheed et al. 2023).

##### 3.2.1.3 VR

###### 3.2.1.3.1 Opportunities

Most VR‐based studies included in this review were conducted in VR supermarkets, which closely replicate the attributes of physical stores and consumer purchasing behavior. In this way, they support the capture of multiple behavioral parameters, such as willingness to buy specific food products and responses to price changes and promotions, without solely relying on self‐reported data. Incorporating motion and eye tracking systems, VR applications record gaze direction, gaze duration, and in‐store movement, revealing how embodied behavioral patterns relate to buying decisions (Schnack et al. 2019, 2020; Xu et al. 2023; Biercewicz et al. 2022; Goedegebure et al. 2020). For instance, Bigné et al. (2016) examined brand choice in a VR supermarket by utilizing such tracking systems to monitor customers’ eye movements, with findings showing that slower eye movements toward a brand within a product category are positively associated with purchase likelihood.

Unlike physical food stores, where external factors and irrelevant stimuli, such as disruptions from other shoppers, interfere with consumers’ decision‐making process, VR settings avoid these distractions, allowing the precise control and repeatability of experimental conditions. In such standardized settings, researchers can isolate parameters and more clearly test behavioral responses to food‐related stimuli. This includes the manipulation of variables, like consumers’ variety seeking tendency. This tendency usually increases in VR stores, as the absence of external distractions enables greater concentration on the shopping process and exploration of more brands and options. This enhanced concentration and engagement contribute to examining with greater accuracy specific outcomes, such as brand choice among a broad range of alternatives. As a result, VR technology is particularly valuable for studying complex behaviors, like variety‐ and information‐seeking, that are difficult to capture in real‐world settings (Bigné et al. 2016; Fang et al. 2021; van Herpen et al. 2016).

Besides variety‐seeking, VR technology demonstrates high adaptability to multiple research contexts, with studies capturing diverse aspects of food consumer behavior. For instance, the simulation of customers’ walking behavior and shelf displays in VR supermarkets enables researchers to examinate the impact of product placement on consumer behavior, avoiding the high cost of changing physical store layouts (van Herpen et al. 2016; Schnack et al. 2019; Violante et al. 2019). Similarly, VR technology can be also applied for pre‐market product testing. For example, Q. J. Wang et al. (2020) used VR as a cost‐effective alternative to assess consumer preferences for coffee presented in different colors. The production and testing of such coffee variations in the physical world be far more expensive and time‐consuming, involving also the risk of unintentionally altering other product attributes, like flavor.

While concerns are raised about the validity of VR‐based results, several comparative studies indicated strong consistency between outcomes of VR experiments and real‐world settings. Indicatively, Lombart et al. (2019) conducted the same survey in VR and physical stores and identified similar consumer perceptions of product quality and purchasing intentions for fruits and vegetables, proving the reliability of VR as an alternative method in consumer research.

Last but not least, the realistic simulation of consumer behavior in VR conditions also enhances users’ perceived telepresence, which refers to the «sense of being». This increased telepresence stimulates users’ enthusiasm and engagement, making VR experiences more enjoyable (Lombart et al. 2020; Han et al. 2020; Andersen et al. 2019; Schnack et al. 2019). Such heightened immersion could provide valuable opportunities in food retail, enriching consumers’ purchasing experience and offering retailers deeper insights into consumer behavior.

###### 3.2.1.3.2 Challenges

Although findings from VR‐based and real‐world experiments often show consistency, some behavioral mismatches remain. The most frequently discussed limitation is that participants in VR typically do not spend real money. This lack of financial constraints may lead to overspending or stronger reactions to promotions. Thus, tracking food expenditures in VR simulations may produce unrealistic outcomes, for example when assessing the impact of price or promotional stimuli on consumer choices (Lombart et al. 2020; van Herpen et al. 2016; Payne Riches et al. 2019). Another mismatch is that users’ heightened sense of being observed in VR may lead to the adoption of socially desirable behaviors, such as purchasing more local food products (van Herpen et al. 2016).

In addition to these behavioral inconsistencies, the accuracy of results is further undermined to an extent, as VR cannot yet provide a fully immersive sensory experience to users. A key limitation is the lack of tactile sensation, which is crucial in assessing food quality and strongly influences purchase decisions, especially for fresh products, like fruits and vegetables (Lombart et al. 2019). While the visual assessment of products is possible in VR settings, some technological constraints remain. These include blurred product images or difficulties in product rotation, hindering realistic simulations and preventing consumers to clearly evaluate food products (Schnack et al. 2019; Alba‐Martínez et al. 2024; Violante et al. 2019). Overcoming these obstacles requires technological advancement. However, progress is constrained by the already high costs of initial investment in VR equipment, making further improvements challenging (Lombart et al. 2019).

From consumers’ perspective, users’ unfamiliarity with VR technology discourages them from participating in VR experiences and increases drop‐out rates in experiments (Fang et al. 2021). This lack of experience can also influence the reliability of results. For instance, when measuring purchasing time, unfamiliar users, facing difficulties in technological use, tend to need longer time to complete shopping tasks than in real‐world settings (Xu et al. 2021; Siegrist et al. 2019). Considering the crucial role of purchasing time in shaping several aspects of consumer behavior, this mismatch raises the risk of misinterpreting results.

##### 3.2.1.4 Neuroscience Tools

###### 3.2.1.4.1 Opportunities

The integration of sensor‐based technologies, particularly neuroscience tools, in consumer research enhances objectivity compared to self‐reported data of traditional approaches, providing real‐time physiological responses to food‐related stimuli. Electroencephalography (EEG) captures brainwave activity on the scalp, offering directly insights into attention and emotional valence, which are central to purchasing behavior. Biercewicz et al. (2024) used EEG in a VR supermarket and showed that higher arousal records were associated with longer purchasing time, underlining the value of EEG in detecting subtle emotional states that influence shopping behavior.

Functional magnetic resonance imaging (fMRI) measures localized brain activation by recording changes in blood flow, allowing researchers to detect which brain areas are activated and involved in consumer decision‐making process. van der Laan et al. (2022) applied fMRI in a VR supermarket to understand consumer responses to promotional stimuli. The results showed that the ventromedial prefrontal cortex (vmPFC), a brain region associated with positive emotions (e.g. reward and value processing), was strongly activated, when participants were exposed to products with a hedonic value, such as chocolate. In contrast, health options like spinach generated weaker and less consistent neural activity. This suggests that the brain system is more responsive to hedonic than health‐related information, indicating that advertisements emphasizing pleasure and taste are more effective. Overall, such tools go beyond traditional research, explaining subconscious emotional drivers of consumer behavior.

###### 3.2.1.4.2 Challenges

Despite these benefits, neuroscience tools are a costly research alternative, requiring specialized facilities and equipment, which limits their use in large samples and thus generalizability of findings. Limitations also remain in data interpretation. EEG and fMRI generate complex datasets, requiring advanced analytical techniques and expertness to extract meaningful insights.

#### 3.2.2 Parameters of Consumer Behavior Assessed

##### 3.2.2.1 Product Perceptions

**FIGURE 7:**
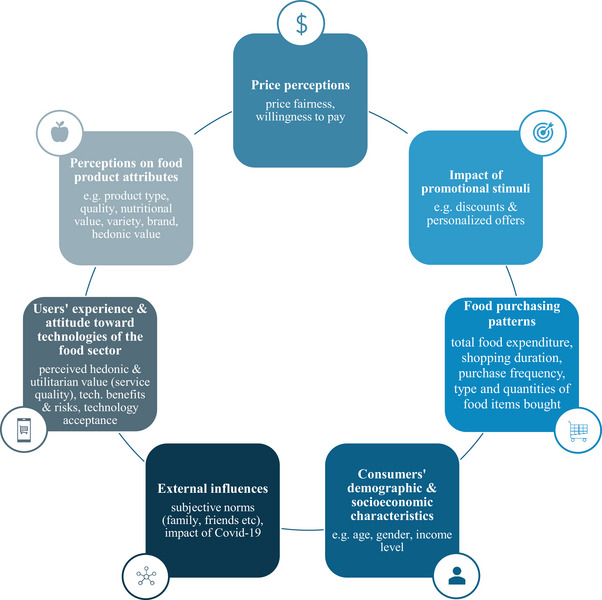
Dimensions of consumer behavior examined in the 159 included articles.

As depicted in Figure 7, one of the key variables, that has been extensively examined through technological tools, includes consumers’ perceptions on food product attributes. More than one‐third of the articles investigated perceived product quality (Torrico et al. 2021; Y. Chen and Zhang 2022; Mishra and Singh 2018), confirming its strong impact on purchasing intentions, particularly for fresh products, like fruits and vegetables. Key quality indicators, including appearance, lifespan, texture, and taste (Lombart et al. 2019), are closely associated with the hedonic value of food products, thus significantly driving buying decisions (Siegrist et al. 2019; Taghikhah et al. 2021). Emerging technologies, particularly SMA, identify large‐scale patterns in product quality perceptions by analyzing consumer reviews (Fisk et al. 2024), while VR allows the controlled manipulation of product attributes and observation of consumers’ responses to these modifications. For example, Q. J. Wang et al. (2020) showed that altering coffee color in VR significantly changed the perceived creaminess, highlighting that consumer reactions can be tested in VR environments, without altering the physical product.

Another important attribute, examined in almost half of the studies, relates to the perceived nutritional value and healthiness of food products, strongly influencing consumer choices. Especially during the pandemic COVID‐19, heightened health awareness resulted in the increase of social media posts and comments addressing nutrition and hygiene, providing researchers rich material to capture these evolving concerns (Brandtner et al. 2021).

Besides health considerations, SMA, particularly Twitter analytics, proved valuable in uncovering perceptions of food products’ eco‐friendliness at large‐scale, as online discussions on sustainability have significantly increased (Brzustewicz and Singh 2021). A big data study analyzing 43,724 tweets indicated that while plant‐based diets and rising environmental concerns dominate discussions, scepticism toward organic claims remains (Singh and Glińska‐Neweś 2022).

The impact of product type on consumer behavior was also explored, with findings showing that consumers tend to adopt different behaviors depending on the food category. For instance, the consumption of certain categories is more strongly driven by hedonic than health motivations. Emerging tools, such as VR experiments, facilitate these comparisons by simulating different product types, while sensory tools like EEG capture how positive emotional responses are more intense for several categories. These include for example fast food and baked foods, indicating the hedonic motivations of such choices (Biercewicz et al. 2024).

Another factor playing a vital role in the decision‐making process is product variety (Tian et al. 2018; Liashenko et al. 2021; Brandtner et al. 2021), strongly reflected in variety seeking behavior. This seeking tendency can be effectively measured through VR simulations, as previously discussed (van Herpen et al. 2016; Bigné et al. 2016). VR can be also effectively used to assess the impact of product placement on consumer behavior. Ploydanai et al. (2017) indicated through a VR‐based experiment that shorter shelves in a non‐parallel orientation led consumers to examine and purchase more products compared to longer shelves. Although VR enables the cost‐effective design and testing of different food store layouts, the influence of product placement on consumer decisions was examined in a relatively small number of articles (Schnack et al. 2020; Ploydanai et al. 2017)

Lastly, brand perception was widely examined through immersive tools, as well as social media and other digital sources that provide rich data on consumers’ brand selection patterns. Many famous food companies maintain digital platforms that allow customers to share feedback, offering valuable insights into the overall potential of a food brand (Lahindah and Diryana Sudirman 2023; Ju 2024).

##### 3.2.2.2 Price Perceptions

Perceived price fairness, strongly determined by perceived food quality, is a key predictor of consumers’ WTP for food products and services offered at specific prices, which in turn drives actual purchase decisions. Online reviews often capture consumer perceptions of price fairness across product categories, which is particularly useful in consumer research. Instead of relying on traditional models, ML‐based systems can further assess price effects more precisely. S. S. Chen et al. (2021) analyzed over 1.2 million supermarket transactions in Germany with a neural network model that estimated how sensitive each shopper was to price changes. The results showed that even small price changes in categories such as vegetables and meat significantly altered purchasing likelihood.

##### 3.2.2.3 Impact of Promotional Stimuli

The impact of promotional stimuli is equally important in shaping consumer behavior and has therefore been examined through emerging tools. The absence of external distractions in VR experiments allows consumers to focus more on the shopping process and notice promotional signs, which enables researchers to track consumer reactions to different marketing strategies and understand their effectiveness (van Herpen et al. 2016).

Sensory tools emerge also as an effective research alternative to explore the influence of promotional approaches. Hakim et al. (2021) recorded and compared EEG signals with self‐reports, while participants viewed distinct food advertisements. Findings indicated that the neural signal of frontal asymmetry, a marker of emotional valence, could be used to predict product preferences more accurately than questionnaire responses, while also providing deeper insights into subconscious dimensions that self‐reported data cannot reflect.

##### 3.2.2.4 Purchasing Behavior Metrics

All the above‐mentioned factors examined shape specific food purchasing patterns, which have been effectively captured with technological tools. Transactional records from food stores objectively capture not only the type of food goods bought and purchase frequency, but also preferred discounted items. This contributes to precisely understanding the effectiveness of marketing strategies (Nelson et al. 2019; Langen and Huber 2023). In addition, transactional analytics can identify the quantity purchased within a specific food category and total expenditures (S. S. Chen et al. 2021; Alawadh and Barnawi 2024). In contrast, VR simulations may overestimate food expenditures, as participants usually do not spend real money and thus tend to overspend. However, they are particularly effective in capturing other important buying metrics, such as purchasing duration. This can be measured by monitoring the time needed to complete tasks and the time users spend observing or searching for specific food items (Blitstein et al. 2020; Blom et al. 2021; Xu et al. 2021).

##### 3.2.2.5 Impact of External Influences

External influences, reflected in subjective social norms, play a vital role in shaping consumer behavior. Stimuli from close circles (family, friends) and from broader social environment (media, government) strongly affect not only food preferences, but also consumers’ willingness to use emerging technologies in food retail (Goedegebure et al. 2020; B. Wang et al. 2023; Pindado and Barrena 2021; Kang and Namkung 2019; Lee et al. 2021). Among these external factors, COVID‐19 was particularly influential in recent years, leading to the digital transformation of the food sector and to significant changes in consumer expectations (Brzustewicz and Singh 2021; Jang et al. 2022b). These shifts were strongly reflected in social media discussions, where consumers expressed increasing interest in food delivery, digital applications, food safety, hygiene, and nutritional quality, during the COVID‐19 period. Such online material was valuable, providing real‐time insights to help retailers adapt to global market changes and evolving consumer demands (Ju 2024; X. Li et al. 2023; Brandtner et al. 2021; Zhang et al. 2021; Jang et al. 2022b).

##### 3.2.2.6 Technology Adoption Factors

Besides using emerging technologies to understand how all these aspects shape food consumer behavior, it is also essential to assess consumers’ intention to adopt these tools, as this adoption is a prerequisite for their effective integration in consumer behavior research. The literature highlighted two main drivers of technology adoption, particularly the hedonic and utilitarian value of technologies.

Regarding the utilitarian aspect, the perceived ease of use and usefulness of new technologies significantly increase individuals’ intention to use them, as confirmed by studies exploring attitudes towards personalized nutrition and food applications (Kang and Namkung 2019; Su, Nguyen, et al. 2022; Berezowska et al. 2015). The quality of digital services strongly shapes this utilitarian value. Dimensions such as timeliness, convenience, pleasantness, easy navigation, and reliability appear very frequently in consumer digital communities, as revealed by several SMA‐based studies, playing a crucial role in consumer intentions to use online food retail services (Su, Nguyen, et al. 2022; S. Chen et al. 2023). Utilitarian value not only enhances customers’ trust and willingness to share information on eating habits and food preferences, but also increases perceived behavioral control. This behavioral control reflects users’ confidence in successfully adopting new technologies (Woodall and Hollis 2024).

Besides the utilitarian aspect, consumers’ intention for technology adoption is equally determined by perceived hedonic value of emerging technologies. Enjoyment and playfulness increase the willingness to adopt tools, such as innovative nutrition platforms, personalized service‐bots, or immersive VR applications, which simultaneously generate valuable consumer data (Yoo et al. 2022; Santiago et al. 2024; Kang and Namkung 2019; Peng et al. 2016). Perceived telepresence in VR environments also enhances users’ engagement, further increasing the likelihood of VR adoption in food retail (Schnack et al. 2019).

Besides perceived advantages, risks, particularly concerns about data security and privacy, also significantly predict technology adoption (Berezowska et al. 2015; Su, Nguyen, et al. 2022). Kang and Namkung (2019) highlighted that higher perceived privacy risks reduce the intention to use personalized food platforms, underlying the importance of enhancing consumers’ sense of safety and trust. Besides trust, technology acceptance is also significantly determined by technology readiness, reflecting individuals’ mental preparedness and willingness to embrace a new technology. This readiness is enhanced by personality attributes, like innovativeness and optimism, but hindered by discomfort and insecurity (Han et al. 2020; Sung and Jeon 2020).

##### 3.2.2.7 Demographic and Socioeconomic Attributes

All the above‐mentioned parameters shaping food consumer behavior and attitudes towards technologies of the food sector are strongly determined by demographic and socioeconomic factors. Age is among the most significant determinants of technology adoption, with older people usually expressing difficulty in using technologies. This discourages them from technology adoption (de‐Magistris et al. 2022; Song and Kim 2022). At the same time, perceived behavioral control in technological use is also influenced by educational level, as higher education often facilitates greater confidence in using technological tools (Rasheed et al. 2023; Kang and Namkung 2019).

Regarding food consumer behavior, some studies utilized digital tools to assess the impact of demographic and socioeconomic attributes on food preferences and buying tendencies (Taghikhah et al. 2021; Shen et al. 2021; Boccia and Tohidi 2024). Nelson et al. (2019) evaluated through transactional analytics the impact of income level on consumers’ food purchasing intentions for organic products. The results showed that while wealthier households bought more organic products, particularly fruits, lower and middle‐income households responded more to price reductions. Similarly, Y. Chen and Zhang (2022) used Twitter posts as a data source to identify differences in attitudes towards alternative meat products between men and women. Findings highlighted a stronger preference for plant‐based alternatives among women. However, besides gender information, researchers could not access further demographic and socioeconomic details for a more comprehensive evaluation of consumer behavior. Notably, this reflects a broader limitation of data‐driven food research. Privacy and ethical constraints often restrict access to demographic details, making it difficult to identify correlations between socioeconomic attributes and consumer food preferences. Thus, most SMA‐based studies, or surveys using big data or transactional analytics, did not examine such characteristics (Green et al. 2020; Zhang et al. 2021; Y. Chen and Zhang 2022).

#### 3.2.3 Food Categories Examined

**FIGURE 8:**
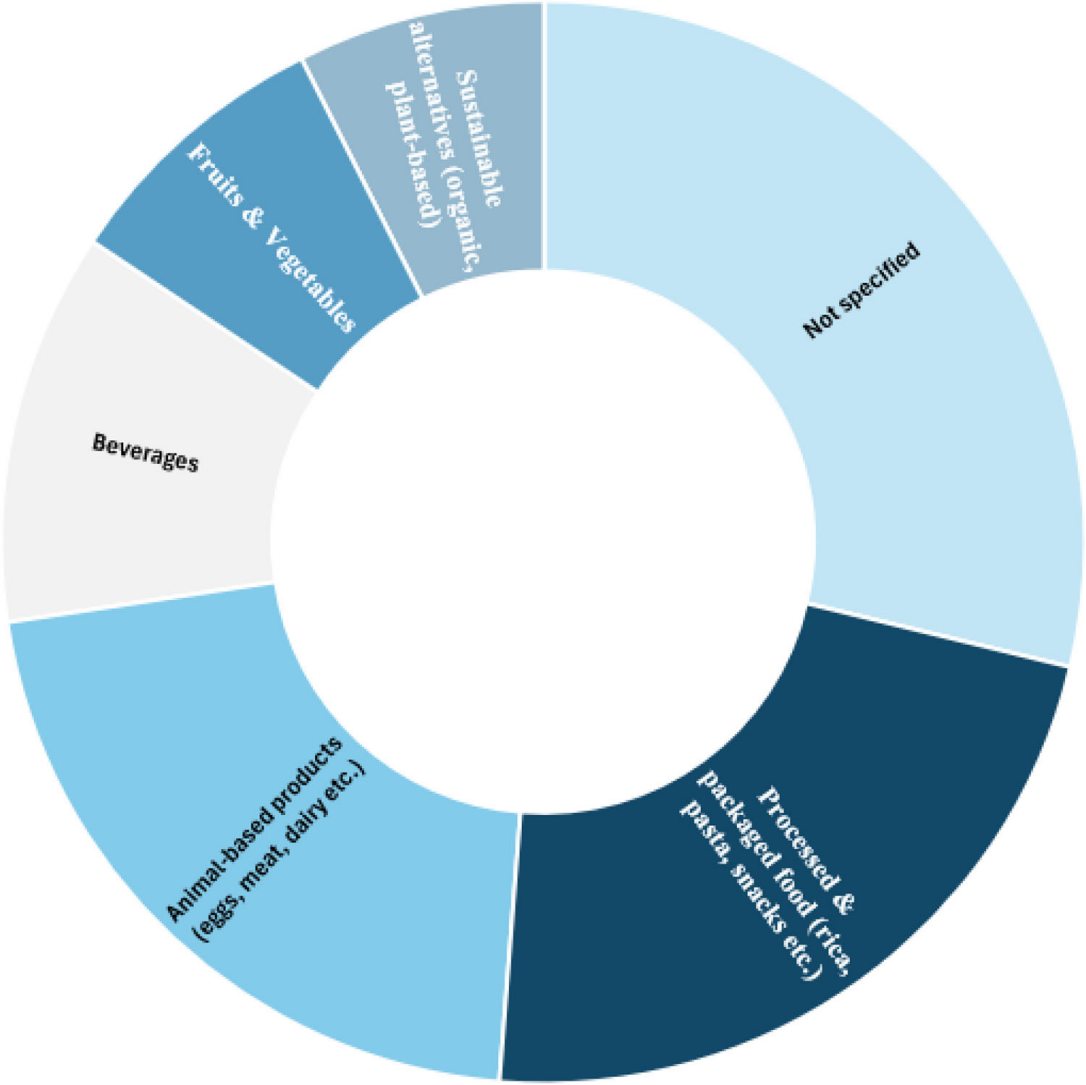
Distribution of the included articles according to the food types toward which consumer behavior was assessed through emerging technologies.

After outlining the main drivers of consumer behavior, this section provides an overview of the food categories toward which consumer attitudes were captured. As presented in Figure 8, the majority of included studies (66 articles) did not focus on a single food type but instead adopted a more general approach, examining broader interactions with food. For example, these studies explored consumer attitudes toward food services, or consumer behavior in relation to emerging technologies in the food sector, such as service‐bots, without focusing on a specific food type.

However, a significant percentage focused on assessing consumer preferences for certain food categories. Among them, the most frequently examined food type includes processed and packaged foods (52 articles), such as rice, pasta, cereals, and snacks, which is likely explained by the convenience and widespread consumption of such goods. A comparable research interest has been directed in the assessment of consumers’ attitude toward animal‐based products (49 articles), including eggs, dairy, meat, and seafood products, most likely due to their high protein content, which makes them central to many diets.

Subsequently, a considerable but smaller number of articles (27 articles) used digital technologies to capture consumer preferences for a wide range of beverages, including both alcoholic and non‐alcoholic products, such as coffee, juices, and tea. Consumer preferences for fruits and vegetables were also captured in several articles through emerging technologies. Last but not least, another part of research focused on sustainability, using technological tools, such as SMA, to assess attitudes toward organic products and plant‐based food alternatives.

### 3.3 Information on the Methodology of the Articles

After discussing the main thematic foci of the retrieved literature, this section provides detailed information about the methodological approaches followed in the reviewed papers.

#### 3.3.1 Theoretical Frameworks of Consumer Behavior

Established theoretical frameworks, including theories and models of consumer behavior, are central to interpreting how various behavioral parameters interact with each other and shape consumer choices. However, a considerable number of articles did not integrate such frameworks but focused instead on developing and applying advanced technology‐based approaches. Indicative examples include highly immersive VR simulations for testing consumer scenarios or analytical techniques like ML or NLP for interpreting large‐scale and complex datasets, both of which represent highly complex and technologically demanding tasks. Because of their demanding nature, the effective use of these methods required particular emphasis on technological aspects, thus reducing reliance on established theoretical frameworks. Nevertheless, the adoption of such advanced tools offered richer and larger‐scale insights into food‐related behavior that would have been difficult to obtain through conventional questionnaire‐based surveys, typically relying on theoretical models of consumer behavior.

However, 25% of the articles incorporated theoretical frameworks, primarily technology adoption models and behavioral intention theories, to assess consumers’ willingness to use emerging technologies that simultaneously facilitate data collection. Technology Acceptance Model (TAM) appeared most frequently (nine articles), highlighting how perceived usefulness and ease of use of emerging technologies, such as personalized food and nutrition applications, strongly predict technology adoption (Kang and Namkung 2019; Su, Nguyen, et al. 2022; Dias et al. 2022). Similarly, theory of planned behavior (TPB) was also frequently used (seven articles), showing how attitudes, subjective norms, and perceived behavioral control determine consumer intentions to use data‐driven technologies within the food sector (Haruyama and Hidaka 2024; Peruzzini et al. 2023). Risk‐benefit evaluation models, particularly privacy calculus theory (PCT), proved valuable as well. Specifically, PCT explains how consumers balance perceived benefits of using personalized food and nutrition platforms or other data‐driven technologies against concerns about privacy and data security risks (Kang and Namkung 2019; Berezowska et al. 2015).

Besides relying on such frameworks to explain drivers of technology adoption, several behavioral models were also applied for interpreting food consumption tendencies. By linking external food‐related stimuli (e.g. marketing approaches) with internal cognitive and emotional responses to these stimuli, the Stimulus‐Organism‐Response (SOR) model was particularly effective in providing more comprehensive understanding of food‐related decision‐making. Moreover, considering the major influence of service attributes on food consumer behavior, several studies combined SMA with established service quality models (SERVQUAL) for a deeper behavioral evaluation. For instance, S. Chen et al. (2023) analyzed and interpreted customer reviews on fresh food logistics using the constructs of SERVQUAL as evaluation indices. Both SERVQUAL and Service Performance Model emerged as key tools for assessing how various factors, such as reliability, tangibility, responsiveness, security, and empathy, shape perceptions on service quality, and how these perceptions in turn determine consumer experiences and behavioral outcomes (S. Chen et al. 2023; Violante et al. 2019; Su, Nguyen, et al. 2022).

#### 3.3.2 Sample Attributes

##### 3.3.2.1 Sample Size

Regarding the sample attributes of studies, for an almost negligible minority of studies (*n* = 8), no sample size was reported (Gadzalo et al. 2020; Glogovețan et al. 2023; Timotijevic et al. 2022). The remaining 151 articles collected consumer data on food preferences and buying patterns, with considerably varying sample sizes, depending on the technology used. Studies based on data‐driven technologies, such as SMA or transactional analytics, typically used massive datasets, often exceeding 10,000 consumer records and sometimes reaching hundreds of thousands or even millions (Izang et al. 2019; Brandtner et al. 2021; Fatemi et al. 2023). For instance, aiming to segment shoppers and identify which food products are mainly purchased together, Alawadh and Barnawi (2024) analyzed over 1,000,000 transactions from 29,356 loyalty customers of a supermarket chain, a sample size that would not be accessible through conventional questionnaire‐based surveys. However, such records (transactions or consumer reviews) do not necessarily correspond to unique consumers, as a single user may contribute more entries.

In contrast, experiments based on VR or sensory technologies, like EEG, involve far smaller samples, typically between 30 and 150 participants, with only few exceptions. This can be explained by the nature of such studies, as they are mainly conducted in controlled laboratory settings, where participants are provided with specialized equipment and detailed instructions to get familiar with technological use. Limited access to the required equipment, as well as the time and spatial demands, restricts sample size.

Although a clear pattern emerged with large samples in data‐driven and smaller ones in VR or sensor‐based experiments, ML showed particular adaptability to research contexts. Specifically, ML algorithms were effectively applied to both large‐scale datasets, often exceeding 10,000 records from social media or transactions (Singh and Glińska‐Neweś 2022; X. Li et al. 2023; Nelson et al. 2019) and smaller ones (100–1500 records) (Taghikhah et al. 2021; Vindigni et al. 2022; Zhu et al. 2023). This indicates that although it is acknowledged that ML algorithms usually perform better with larger datasets, they remain effective in handling also small‐scale data, providing meaningful insights into consumer behavior that traditional statistical models might overlook.

##### 3.3.2.2 Age Group of Participants

A pattern between participants’ age groups and the type of technology utilized for consumer behavior assessment was observed. Experiments based on VR or sensory technologies involved smaller samples of young participants, primarily university students (van der Laan et al. 2022; Lombart et al. 2019, 2020). This reflects their implementation in controlled environments, often within universities, where students are both accessible and more familiar with technological use. In contrast, in many studies using big data or digital sources, did not report participants’ age at all, as demographic details are often unavailable publicly because of privacy and data security restrictions.

##### 3.3.2.3 Geographical Level of Data Collection

**FIGURE 9:**
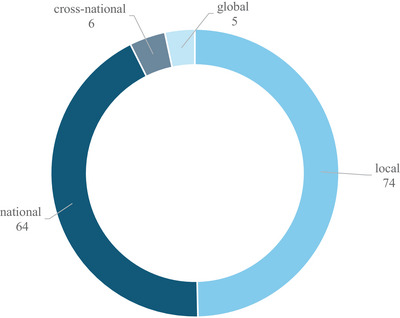
Distribution of the included studies according to the geographical level at which consumer data were collected.

As observed in Figure 9, most articles collected consumer data at a local or national level. This was particularly obvious in VR‐based experiments, as the need of physical presence and specialized equipment restricted the geographical level of data collection. Similarly, articles using SMA mostly relied on national data too. Indicatively, X. Li et al. (2023) analyzed UK supermarkets tweets during the first COVID‐19 lockdown, with sentiment strength detection (SentiStrength). SentiStrength is a lexicon‐based tool that estimates the intensity of positive and negative sentiment in short texts like social media posts. This approach showed how such automated methods, typically integrated during using SMA, depend on consistent linguistic cues and perform better when all data are in a single language, partly explaining the predominance of national‐level data in SMA‐based studies.

Except for SMA, many studies utilizing other emerging technologies have been conducted at a national level too (Alawadh and Barnawi 2024; Taghikhah et al. 2021), thereby overlooking cross‐national comparisons that are particularly important given the strong influence of cultural contexts on food consumer behavior. This is notable, as big data approaches eliminate the need for physical presence and thus facilitate research at a broader geographical scale. The limited number of articles taking advantage of this opportunity may reflect limitations, such as linguistic barriers, as previously described, and research scopes often focused on local products or other parameters, rather than on the comparison of food consumption patterns across different geographical contexts.

#### 3.3.3 Methodological Approach (Quantitative or Qualitative)

The reviewed studies have been classified as quantitative, qualitative, or mixed according to their methodological approach. However, many studies combined qualitative inputs with quantitative processing, making the boundaries between these approaches less clear. To ensure consistency and comparability, categorization was therefore based on the methods of data analysis and interpretation, rather than on the inherent type of collected data. For instance, while textual reviews on food products and services are qualitative in origin, they were mainly processed and interpreted in a totally automated way. The application of ML algorithms, topic modeling techniques, automated word‐frequency, and NLP‐based sentiment analysis in large datasets are some examples proving the fundamentally quantitative character of most SMA‐based studies (Brandtner et al. 2021; X. Li et al. 2023; Brzustewicz and Singh 2021). Similarly, VR‐based experiments, despite providing deep behavioral insights through motion‐ and eye‐tracking systems, they were generally categorized as quantitative too, since consumer data were captured in a numeric form via automated digital tracking and analyzed with statistical techniques (Siegrist et al. 2019; Goedegebure et al. 2020). However, in many other cases, the collected data were originally numeric, such as transactional records providing structured information on food spending patterns (Alawadh and Barnawi 2024; Morris et al. 2020).

Based on this classification approach, approximately 85% of the reviewed studies were characterized as quantitative. Common types of quantitatively analyzed data include: a) buying metrics captured in VR‐based experiments or through transactional records (Lombart et al. 2020; Nelson et al. 2019; Alawadh and Barnawi 2024), b) word frequencies and sentiment scores of UGC reflecting consumer perceptions on food product and service attributes (S. Chen et al. 2023; Y. Chen and Zhang 2022), and c) online ratings of food products, stores, and services (Brandtner et al. 2021; Culotta and Cutler 2016).

A smaller number of studies (15 articles) adopted mixed research approaches, for example by combining big data and algorithmic techniques with qualitative methods (e.g. in‐depth interviews). This enabled researchers to obtain complementary behavioral insights into underlying drivers of food consumption patterns identified through quantitative findings (Schnack et al. 2019; Song and Kim 2022; Su, Nguyen‐Phuoc, et al. 2022). Such mixed approaches, providing both robust, objective quantitative data and qualitative behavioral insights, enable a more comprehensive evaluation of consumer behavior.

For instance, Jones‐Garcia et al. (2022) used a “smart bin” equipped with sensor‐based technologies to automatically collect quantitative data on household food waste through image capturing and food weighing. This allowed the objective measurement of the quantity and type of food waste within each household. At the same time, in‐depth interviews were conducted with participants focusing on food practices, consumption habits, perceptions, and feelings regarding food waste. The combination of these qualitative insights with those captured through sensor‐based data both quantified the extent of food waste and revealed the underlying factors behind food waste behavior. Notably, in some cases, the waste recorded by the smart bin differed from participants’ self‐reports, indicating that consumers often underestimate or do not realize how much food they discard. So, the use of such sensor‐based technologies can increase consumers’ awareness, while also offering researchers valuable insights, especially when combined with qualitative approaches.

Lastly, a very limited percent of studies (<5%) adopted an exclusively qualitative approach, such as manual reading and thematic analysis of UGC (Ariyasriwatana and Quiroga 2016; J. Li and Hu 2021). Τhe in‐depth reading and examination of consumer views offer deeper insights into drivers of food‐related behavioral outcomes and can help address limitations, such as reduced accuracy of automated NLP in detecting irony. However, this approach involves risks of bias, particularly due to the researchers’ subjective interpretation.

#### 3.3.4 Data Analysis Techniques

The most common data analysis techniques applied in the reviewed articles include:

- Correlation analysis to assess the strength of associations between variables of food consumption behavior (Taghikhah et al. 2021; Q. J. Wang et al. 2020; Xu et al. 2021),
- ANOVA and *t*‐tests to compare mean values across several consumer groups and assess whether these differences are statistically significant (Alba‐Martínez et al. 2024; de‐Magistris et al. 2022; Torrico et al. 2021; Frank et al. 2024),
- Regression analysis to predict specific consumer behavioral outcomes from independent variables (Dias et al. 2022; Arrazat et al. 2023),
- Mediation analysis to test whether the relationship between an independent and a dependent variable is determined by a third variable (mediator) (Dias et al. 2022; Habib and Hamadneh 2021),
- Confirmatory factor analysis (CFA) to test whether the hypothesized relationships among consumer behavioral variables are confirmed in the collected dataset (Han et al. 2020; Kang and Namkung 2019),
- Exploratory factor analysis (EFA), with principal component analysis (PCA) being the most often used exploratory method, to reduce the volume of collected data by summarizing them into a smaller number of principal components that shape consumer behavior (Shyu et al. 2023; van Herpen et al. 2016; Su, Nguyen, et al. 2022; Torrico et al. 2021),
- Structural equation model (SEM) to analyze complex relationships between multiple variables and understand how behavioral parameters interact (Su, Nguyen‐Phuoc, et al. 2022; Sung and Jeon 2020; Nunkoo et al. 2024),

These methods are widely recognized for analyzing consumer data and understanding food consumption behaviors. Although commonly applied with traditional statistical tools in smaller‐scale studies (e.g. questionnaire‐based surveys), some techniques, such as regression, classification, and cluster analysis, can be also implemented in more advanced ways, typically through ML algorithms to analyze larger or more complex datasets. For example, when handling large‐scale, textual datasets like UCG, automation is essential not only due to volume, but also because of unstructured data type.

In this context, SMA‐based studies often integrated automated techniques, such as Term Frequency—Inverse Document Frequency (TF‐IDF) analysis to quantify the importance of specific terms by highlighting words that appear frequently in a given text but rarely across the entire dataset. To interpret the contextual meaning of the most important terms identified, TF‐IDF analysis was in most cases followed by sentiment analysis. Sentiment analysis aims to assess the emotional tone of food‐related UGC (e.g. social media posts, online reviews), classifying it as positive, negative, or neutral (Y. Chen and Zhang 2022; S. Chen et al. 2023; Mishra and Singh 2018; Brandtner et al. 2021; Brzustewicz and Singh 2021; Jang et al. 2022a, 2022b).

To improve automation in sentiment analysis, many articles integrated supervised ML techniques, particularly classification algorithms that assign the collected data into predefined categories. Among them, Support Vector Machine (SVM) was widely used to separate data into classes by constructing a boundary (hyperplane) that maximizes the distance between them (Nguyen et al. 2021). For example, Y. Chen and Zhang (2022) classified more than 42,000 posts on alternative meat into five predetermined sentiment categories through SVM, showing how this algorithm can effectively distinguish consumer opinions. More advanced models, such as Extreme Gradient Boosting (XGBoost), have been also applied in several articles. XGBoost constructs decision trees subsequently, with each one correcting errors of the previous, to improve accuracy.

Unlike supervised classification techniques that sort data into predefined groups, unsupervised ML methods have been used for cluster analysis to identify similar clusters without requiring prior labelling. Clustering algorithms are particularly valuable for market segmentation, grouping together customers with shared characteristics and food preferences, without depending on predefined clusters. For example, density‐based spatial clustering of applications with noise (DBSCAN) uncovers market segments in complex datasets by detecting groups of data points that are close to each other and treating points that do not fit into any cluster as noise (Taghikhah et al. 2021; Pindado and Barrena 2021).

Last but not least, another unsupervised ML technique frequently applied in the studies is topic modeling, specifically Latent Dirichlet Allocation (LDA). LDA is a probabilistic algorithm that identifies thematic topics within large textual datasets by grouping together words that co‐occur (Choi et al. 2024). For instance, Brzustewicz and Singh (2021) used LDA to analyze online food reviews on vegetarian restaurants and extracted topics like food quality, service quality, and atmosphere, demonstrating how LDA can reveal hidden factors shaping consumer attitudes towards vegetarian restaurants

In conclusion, many articles went beyond traditional statistical tools by incorporating ML and other automated techniques which are essential for handling large‐scale, unstructured data. This is particularly important today, as vast amounts of consumer data, richer in content and variety, are increasingly accessible and can be systematically analyzed only through automation.

#### 3.3.5 Comparison of Technology‐Based With Traditional Research Approaches

While many researchers adopted innovative technologies to capture food‐related behavioral patterns, only a few of them directly compared their potential as research tools with traditional research methods. For instance, Xu et al. (2021) conducted a comparative study where participants evaluated the healthiness of different cereals in both a physical environment and a VR one designed to be identical. Results indicated that consumer perceptions of cereal healthiness and information‐seeking behavior were similar in both environments, proving the validity of VR for capturing food‐related behavior. While in this experiment participants were exposed to actual food products, VR‐based methods were also evaluated against other traditional approaches, such as the photo‐enhanced imaginative condition (PIC). PIC is a method where participants view a static image and imagine themselves in that context. Andersen et al. (2019) demonstrated that although PIC can capture how external factors influence consumer reactions, it lacks the interactive and immersive character of VR. Specifically, in their study, only VR triggered significantly stronger desire for specific beverages in a beach setting, showing its ability to provide more realistic simulations of consumption contexts and behaviors This was further confirmed by Fang et al. (2021), who proved that preferences for dairy products expressed in VR experiments were closer to actual purchases than those from PIC‐based studies, demonstrating VR's potential to reduce hypothetical bias.

Besides VR, technology‐based research methods, such as the use of data‐driven technologies for capturing food‐related consumer behavior, have been compared with questionnaire‐based surveys. For instance, Vepsäläinen et al. (2022) compared grocery transactional records with self‐reported data using the same sample, aiming to identify whether the food purchased is also consumed. Results suggested a strong similarity between transaction and self‐reported data, showing that grocery purchase data can be used as a validated indicator of actual food consumption behavior. Lastly, a limited number of studies compared ML techniques against traditional statistical methods and econometric models to determine which approach is more accurate and effective in predicting food purchasing behaviors. Findings suggest that combining advanced with traditional methods offers richer and more reliable insights into food‐related patterns (Donnelly et al. 2021; Nelson et al. 2019).

Besides providing a more comprehensive and deep evaluation of consumer behavior, the integration of diverse research methodologies also enhances research reliability. The direct comparison of technology‐driven with traditional approaches is crucial for assessing whether and how such methodological differences affect outcomes in consumer behavior research. In this way, the reliability and limitations of each technology approach can be outlined, enhancing overall robustness. However, the number of consumer studies combining traditional with technology‐driven methods in both data collection and analysis remains notably limited, which is an important literature gap.

## 4 Answers to RQs, Implications and Future Research Agenda

After the thorough evaluation of the above‐mentioned criteria, this section summarizes the main findings, addressing each RQ explicitly.

### 4.1 Answers to RQs

#### 4.1.1 RQ1: Which Behavioral Dimensions of Food Consumption Can be Captured via Emerging Technologies and How Can the Collected Data be Analyzed and Interpreted?

Emerging technologies capture a wide range of behavioral factors, from perceptions and emotions to actual purchasing behaviors. VR enables the manipulation of variables, such as product variety, shelf placement, marketing cues, and sensory attributes, and the precise observation of embodied consumer responses. Neuroscience tools further capture emotional reactions to these stimuli, while SMA offer insights into consumer perceptions (e.g. product perceptions, brand preference, and price sensitivity) and identify emerging food trends. Transactional records, in turn, reflect the purchasing outcomes of all these perceptions and emotions, including food expenditures, preferred items, and purchase frequency.

Large‐scale and often unstructured data, collected by data‐driven tools, can be automatically processed through advanced techniques that go beyond the capacity of traditional statistical models. ML methods, such as clustering algorithms, segment consumer groups based on shared characteristics, while text‐mining approaches, particularly TF‐IDF and NLP‐based sentiment analysis, automatically interpret textual data (e.g. social media posts) to uncover large‐scale patterns and food preferences.

#### 4.1.2 RQ2: What Are the Key Benefits and Challenges of Using These Technologies in Food‐Related Consumer Research Compared to Traditional Research Methods?

Data‐driven technologies (SMA, transactional analytics, and ML) offer clear advantages compared to traditional methods, providing large‐scale, updated consumer data, and enabling the automated analysis of complex and unstructured information with improved efficiency. However, important challenges remain, including reduced demographic details, dependence on high‐quality datasets and overfitting risks in ML applications, significant computational requirements, high methodological complexity, and often limited of explanatory depth. In contrast, VR tools provide deeper behavioral insights, especially compared to self‐reported data by simulating purchasing experiences in controlled and realistic settings. Yet, their validity is limited by reduced sensory immersion, which can result in behavioral inconsistencies. Finally, neuroscience tools, while capturing subconscious drivers of food‐related behaviors that are inaccessible through self‐reported data, their adoption is limited due to high costs and technical requirements.

#### 4.1.3 RQ3: Based on These Challenges and Methodological Gaps, What Are the Future Research Directions?

Besides technological limitations, such as reduced sensory immersion in VR or limited accuracy in interpreting some linguistic nuances through NLP tools (e.g. sarcasm or irony), methodological limitations also remain. The fundamentally quantitative character of studies based on data‐driven technologies, combined with limited demographic information and the rare use of established theoretical frameworks, reduces explanatory depth. Moreover, only a few studies compared technology‐based findings with outcomes of traditional research methods. Without systematic comparisons, the validity and consistency of technology‐driven outcomes remain uncertain, especially given the technological barriers previously discussed and the continuously evolving nature of recording tools such as VR and ML. Last but not least, most technology‐driven research relied on local‐ or national‐level datasets, limiting generalizability of findings and overlooking cross‐cultural differences. This is a critical gap in the literature, especially since food consumption patterns are strongly shaped by cultural, social, and economic contexts. These gaps lead to several directions for future research, which are summarized below in Section 4.2.

#### 4.1.4 RQ4: How Do Consumers Perceive the Integration of New Technologies Enabling Consumer Behavior Analysis in the Food Sector?

Consumer intention to adopt emerging technologies that facilitate data collection is shaped by both utilitarian and hedonic values. Perceived ease of use, usefulness, improved service quality (e.g. convenience, reliability, and timeliness) increase acceptance, particularly for digital food services and personalized apps. At the same time, enjoyment, playfulness, and perceived telepresence drive the adoption of immersive technologies in food retail. However, data security and privacy concerns remain a major barrier, especially in interactions with personalized and robotic systems that collect consumer information, making trust a critical prerequisite for consumer engagement.

### 4.2 Implications and Future Research Agenda

Based on these findings, and research and methodological gaps uncovered, the following implications and future research directions are suggested:

- Adopting data‐driven technologies in food‐related consumer research to access large scale, updated and diverse consumer data, and automatically analyze them,
- Integrating immersive technologies in food consumer behavior assessment, as they enable the controlled simulation of purchasing experiences,
- Using neuroscience technologies to extract objective measurements into subconscious, food‐evoked emotions,
- Designing user‐friendly and enjoyable digital systems to stimulate consumers’ engagement and adoption, and reduce drop‐out rates (e.g. in VR experiments),
- Establishing clear regulations about data protection and privacy to enhance consumers’ trust and engagement, especially with personalized systems,
- Encouraging future research on technological advancement to improve algorithmic capabilities and performance, and increase realism of immersive environments,
- Integrating established theoretical frameworks in technology‐based research to better understand and interpret in a structured way the underlying psychological mechanisms of food‐related behaviors,
- Extending the geographical level of data collection in technology‐driven consumer research to capture cross‐cultural differences and global food trends,
- Combining quantitative with qualitative research methods (e.g. focus groups, interviews) to complement large‐scale findings with deeper behavioral insights and demographic links,
- Validating and comparing the outcomes of technology‐based findings (e.g. SMA or VR‐based findings, outputs from ML modes) with traditional research methods to ensure reliability, depth, and account for potential technological limitations.

Lastly, a possible improvement of this systematic review would be the inclusion of industry‐type and designation‐based categorizations to acquire a deeper understanding of the current literature.

## 5 Conclusions

The growing need to understand food consumer behavior, aligned with the limitations of traditional research approaches, typically based on questionnaires, motivates the exploration of alternative research methods. This review highlights how a broad range of emerging technologies can uncover diverse aspects of consumer behavior, from perceptions and food‐evoked emotions to actual purchasing outcomes. Besides outlining the advantages and barriers of each technological approach, it also demonstrates the methodological approaches followed in technology‐based consumer studies. By considering methodological details, technological applications, and consumer dimensions, this review provides a comprehensive perspective on how technological innovations can be effectively leveraged to advance food consumer behavior research. According to its findings, researchers and food retailers can access large‐scale, diverse and updated consumer insights via data‐driven tools (SMA, transactional analytics, big data, and personalized systems), enhance automation and efficiency through ML approaches, and realistically simulate food‐related behaviors in VR settings. At the same time, technological integration benefits consumers too by offering them improved food services and personalized experiences. However, important methodological gaps were uncovered, such as the frequent lack of demographic information, the limited use of established theoretical frameworks, narrow geographical scope, and technological barriers like ML performance. Addressing these issues as proposed in future research agenda will enable deeper and more reliable insights into food consumption patterns. Such insights can in turn support the design of more effective marketing frameworks and the development of food products and services that better reflect consumer expectations and preferences.

## Author Contributions

**Kyriaki Kechri**: investigation, writing – original draft, visualization, methodology, conceptualization. **Christina Kleisiari**: validation, conceptualization, writing – review and editing. **Leonidas Sotirios Kyrgiakos**: validation, conceptualization, methodology. **Marios Vasileiou**: conceptualization, validation, investigation. **Dimitra Despoina Tosiliani**: visualization, conceptualization. **Vasileios Angelopoulos**: conceptualization, visualization. **George Kleftodimos**: conceptualization, writing – review and editing, validation. **George Vlontzos**: conceptualization, supervision, writing – review and editing.

## Conflicts of Interest

The authors declare no conflicts of interest.

## Supporting information




**Supplementary Table**: crf370340‐sup‐0001‐tableS1.docx
