# Supplementary material for: Emerging Technologies for Investigating Food Consumer Behavior: A Systematic Review
Source: Compr Rev Food Sci Food Saf. 2025 Nov 16;24(6):e70340. doi: 10.1111/1541-4337.70340 (PMC12620339; doi:10.1111/1541-4337.70340)
Supplement: Supplementary file 1 — Supplementary Table: crf370340‐sup‐0001‐tableS1.docx [file CRF3-24-e70340-s001.docx]

Table 1: Key features and findings of the reviewed studies

| N | Type of technology applied/ examined | Benefits of technological use | Challenges of technological use | Factors examined | Food type examined | Reference |
| --- | --- | --- | --- | --- | --- | --- |
| 1 | SMA | Updated data, larger sample size, low research cost, eWOM | Challenging data handling | Brand perception (eco-friendliness, healthiness) | Not specified | (Culotta & Cutler, 2016) |
| 2 | VR | Quicker research method, simulation of consumer behavior, motion tracking, adaptability to contexts, better experimental control, telepresence, product testing before releasing | Limited accuracy in the simulation of some variables | Impact of product type, placement, price, promotion, brand, variety seeking, WTP, quantities purchased, money spent, telepresence, demographics | Fruits, vegetables, snacks, dairy | (van Herpen et al., 2016) |
| 3 | VR | Low research cost, better experimental control, digital replication of consumer behavior, motion & eye tracking | Users’ unfamiliarity, need for tech advancement, limited accuracy in simulating some variables | Perceived healthiness & hedonic value, purchasing time, cognitive aspects, telepresence, demographics | Cereals | (Siegrist et al., 2019) |
| 4 | Big Data, SMA | Quicker research method, larger sample size, real-time data | Challenging data handling, security & privacy concerns | Perceived product quality, food waste | Meat | (Mishra & Singh, 2018) |
| 5 | Apps with personalized services | Personalization, enhanced utility value and users’ trust | Privacy & security concerns, technological anxiety | Attitude towards tech applications (personalization, privacy & security concerns, utility & hedonic value, trust, technology acceptance) | Not specified | (Kang & Namkung, 2019) |
| 6 | VR | Simulation of consumer behavior, motion & eye tracking, enhanced telepresence and hedonic value | Users’ unfamiliarity, need for tech advancement, limited accuracy in the simulation of some variables | Impact of product type and brand, variety seeking, buying metrics (quantities bought & purchasing time), demographics | Alcoholic beverages | (Bigné et al., 2016) |
| 7 | VR | Enhanced usefulness, telepresence and hedonic value | Security concerns, perceived risks (product quality), unfamiliarity, limited accuracy in simulating some variables | Users’ experience (usefulness, ease of use, behavioral control, hedonic value, telepresence, technology acceptance), demographics | Not specified | (S. L. Han et al., 2020) |
| 8 | VR | Low research cost, simulation of consumer behavior, motion tracking, enhanced utility value and telepresence | Unfamiliarity, tech limitations, need for tech advancement, limited accuracy in the realistic simulation of some variables | Users’ experience (ease of use, telepresence), demographics | Not specified | (Schnack et al., 2019) |
| 9 | Apps with personalized services | Personalization, improved service quality, utility value, enhanced users’ trust and engagement | Security - privacy concerns, unfamiliarity | Attitude towards tech applications (trust, ease of use & usefulness, privacy concerns, perceived service quality), demographics | Not specified | (Su, Nguyen, et al., 2022) |
| 10 | VR | Better experimental control, simulation of consumer behavior, adaptability to contexts, telepresence, product testing | Lack of sensation of touch, high development cost, limited accuracy in the replication of some variables, tech limitations🡪 need for advancement | Perceived product quality, healthiness, price, buying metrics (quantities bought, purchasing time, money spent), demographics | Fruits & vegetables | (Lombart et al., 2019) |
| 11 | VR | Better experimental control, simulation of consumer behavior, enhanced telepresence and hedonic value | Lack of sensation of touch, limited accuracy in simulating some aspects of consumer behavior, tech limitations | Perceived product quality, price, healthiness, hedonic value, quantities purchased, money spent, demographics | Fruits & vegetables | (Lombart et al., 2020) |
| 12 | Food apps | Enhanced hedonic and utility value | Security and privacy concerns, users’ unfamiliarity | Attitude towards tech applications (trust, utility & hedonic value, security & privacy concerns, subjective norms), demographics | Not specified | (Habib & Hamadneh, 2021) |
| 13 | VR | Low research cost, better experimental control, simulation of consumer behavior, enhanced telepresence & hedonic value, product testing | High development cost, need for tech advancement, limited accuracy in simulating some aspects of consumer behavior | Impact of product type, external influences, telepresence, demographics | Beverages (alcoholic and non-alcoholic), snacks | (Andersen et al., 2019) |
| 14 | Nutrition apps with personalized services | Cost-effective and quicker research method, enhanced utility, hedonic value and healthiness, personalized & educational content | Drop-out rate, privacy & security concerns, users’ unfamiliarity | Healthiness, attitude towards tech applications (ease of use & usefulness, perceived risks, hedonic value), demographics | Not specified | (Peng et al., 2016) |
| 15 | Big Data: online reviews analytics | Low research cost, large-scale and real-time data, predictive ability | Not reported | Product type, perceived service & product quality, variety, price, impact of Covid-19 | Not specified | (Brandtner et al., 2021) |
| 16 | Service-bots | Personalization, improved service quality, usefulness and hedonic value | Lack of human interaction, need for tech advancement, unfamiliarity | Attitude towards robotics (usefulness, technology acceptance, hedonic value, perceived service quality, need for human interaction) demographics | Not specified | (Sung & Jeon, 2020) |
| 17 | Service-bots | Personalization, enhancement of service quality and usefulness | Unfamiliarity, need for tech advancement, lack of human interaction, privacy - security concerns | Attitude towards robotics (utility value, need for human interaction, technology acceptance, privacy issues, service quality), demographics | Not specified | (Song & Kim, 2022) |
| 18 | ML | Large-scale data handling, market segmentation, predictive accuracy | Challenging data handling, overfitting risk | Trust in organic claims, subjective norms, perceptions on product attributes (hedonic value, eco-friendliness, healthiness, quality, price, brand), WTP, buying metrics, demographics | Organic products | (Taghikhah et al., 2021) |
| 19 | VR | Cost effective & quicker research method, accuracy, simulation of consumer behavior, adaptability to contexts, telepresence, personalized experience, utility value | Unfamiliarity, high computational & development cost, need for tech advancement, lack of sensation of touch | Telepresence, hedonic value, service quality, demographics | Not specified | (Violante et al., 2019) |
| 20 | Online reviews analytics, ML | Low research cost, accuracy, large-scale and updated data, personalization | Privacy & security concerns, challenging data handling | Perceived service & product quality, eco-friendliness | Fruits, vegetables, meat, eggs, seafood | (S. Chen et al., 2023) |
| 21 | VR | Simulation of consumer behavior, adaptability to research, better experimental control, enhanced telepresence | Unfamiliarity, tech limitations, need for tech advancement | Price perceptions, WTP, healthiness, telepresence, demographics | Dairy | (Fang et al., 2021) |
| 22 | VR | Low research cost, better experimental control,  adaptability to research contexts, motion tracking, enhanced telepresence | Lack of sensation of touch, limited accuracy in simulating some variables, need for tech advancement, users’ unfamiliarity | Impact of product type, brand, product placement, buying metrics (purchasing time, quantity bought, money spent), demographics | Beverages, snacks, dairy, legume and pulse products | (Schnack et al., 2020) |
| 23 | Big Data: SMA, ML | Accuracy, market segmentation, larger sample size, eWOM | Challenges in data handling, requirement of large datasets, need for tech advancement | Perceived product & service quality, price | Plant-based foodservice products | (Nilashi et al., 2021) |
| 24 | ML, EEG | Objective measurements, predictive ability | High cost, technological limitations & complexity, challenging data handling, need for tech advancement | Impact of promotional stimuli, demographics | Snacks | (Hakim et al., 2021) |
| 25 | SMA | Improved service quality | Not reported | Perceptions on product & service quality in food delivery (ease of use, usefulness, privacy & security issues, trust) | Not specified | (Su, Nguyen-Phuoc, et al., 2022) |
| 26 | Apps with personalized services | Personalization, educational content | Privacy & security concerns | Attitude towards tech applications (privacy & security issues, service quality, trust), demographics | Not specified | (Berezowska et al., 2015) |
| 27 | SMA, ML | Updated & large-scale data, accuracy, eWOM | Not representative sample distribution, challenging handling of unstructured data | Perceived service quality, social influence, impact of Covid-19 | Not specified | (X. Li et al., 2023) |
| 28 | Big Data: SMA, ML | Objectivity, accuracy, large-scale and updated data | Difficulty in data handling, requirement of large datasets, not representative sample distribution | Perceptions on product quality (eco-friendliness, healthiness, hedonic value), trust in organic claims | Organic products | (Singh & Glińska-Neweś, 2022) |
| 29 | Big Data: SMA, ML | Larger sample size, updated and unbiased data compared to interviews | Limited accuracy in the automated interpretation of some linguistic cues | Sustainable consumption (healthiness, eco-friendliness), impact of Covid-19 | Not specified | (Brzustewicz & Singh, 2021) |
| 30 | VR | Cost-effective and quicker research method, better experimental control, simulation of consumer behavior, adaptability to contexts | Users’ unfamiliarity | Perceived product quality, demographics | Beverages | (Q. J. Wang et al., 2020) |
| 31 | Service-bots | Improved service quality, usefulness and hedonic value | Privacy & security concerns, unfamiliarity, lack of human interaction | Attitude towards robotics (ease of use, usefulness, technology acceptance, behavioral control, hedonic value, security & privacy issues, service quality), demographics | Not specified | (Rasheed et al., 2023) |
| 32 | VR | Replication of consumer behavior, telepresence, better experimental control, adaptability to contexts, product testing before releasing | Limited accuracy in the simulation of some examined variables, users’ unfamiliarity | Cognitive aspects, healthiness, purchasing time, users’ experience (telepresence, ease of use), demographics | Cereals | (Xu et al., 2021) |
| 33 | VR | Simulation of consumer behavior, enhanced telepresence | Users’ unfamiliarity, technological limitations | Perceived product quality (hedonic value, healthiness), demographics | Snacks | (Torrico et al., 2021) |
| 34 | Big data: SMA, ML, TL | Higher accuracy (TL), larger sample size, updated data | Not representative sample distribution, unavailability of data on users’ socioeconomic attributes, limited accuracy in interpretating some linguistic cues | Perceptions on product attributes (healthiness, eco-friendliness, trust in organic claims, quality, brand, price), impact of promotion | Plant-based products | (Y. Chen & Zhang, 2022) |
| 35 | Big Data: SMA | Real-time data, larger sample size, cost-effective and quicker research method | Limited accuracy in the automated interpretation of some linguistic cues, need for tech expertness, potential misinformation/ bias, not representative sample distribution | Perceived service quality | Not specified | (He et al., 2018) |
| 36 | Transactional analytics, ML | Large-scale data, predictive accuracy, personalization | Challenging data handling, technological limitations | Price | Fruits, vegetables, dairy, cereals, meat, snacks | (S. S. Chen et al., 2021) |
| 37 | Chatbots, ML: Transfer learning | Personalization, improved service quality (bots) | Need for technological advancement, overfitting risk (ML), requirement of large datasets (TL) | Service quality | Cereals, meat | (Chiu & Chuang, 2021) |
| 38 | Big Data: SMA, ML | Large-scale and updated data, data geolocation - spatial analysis | Not representative sample distribution, limited accuracy in the automated interpretation of linguistic cues, tech complexity | Food trends, social influence (cultural differences) | Not specified | (Pindado & Barrena, 2021) |
| 39 | Big Data, online reviews analytics | eWOM, large-scale data | Need for technological expertness | Perceived product quality (hedonic value, healthiness), environmental awareness, social influence, demographics | Meat | (B. Wang et al., 2023) |
| 40 | VR | Realistic simulation of consumer behavior, motion tracking | Participants’ drop-out rate, users’ unfamiliarity | Social influence, perceived product quality (healthiness), impact of promotional stimuli and brand, trust, demographics | Dairy, meat, beverages, snacks | (Goedegebure et al., 2020) |
| 41 | Big Data, ML | Predictive accuracy | Challenging & time-consuming data handling | Demand, price, quantities purchased | Meat | (Ryu et al., 2020) |
| 42 | ML | Analysis of complex data, predictive accuracy | Need for technological expertness | Perception on NPBT products (price, eco-friendliness, healthiness, quality, trust, risks), cognitive aspects, demographics | Not specified | (Vindigni et al., 2022) |
| 43 | Big Data, SMA | Cost-effective research method, updated data, larger sample size | Not representative sample distribution, limited accuracy in the automated interpretation of some linguistic cues | Perceived healthiness & environmental impact of products, price perceptions, WTP, emotional & cognitive aspects, demographics | Eggs | (Widmar et al., 2020) |
| 44 | Online food apps | eWOM, personalization, improved service quality | Not reported | Attitude towards online purchases (service quality, sustainability, product attributes – healthiness. price), demographics | Seafood | (Sigurdsson et al., 2017) |
| 45 | ML | Market segmentation, accuracy | Technological complexity, challenging data handling | Attitude towards grocery apps, demographics | Not specified | (Salamzadeh et al., 2022) |
| 46 | AI-driven nutrition app with personalized services | Educational content, personalization, utility and hedonic value | Users’ drop-out rate | Attitude towards nutrition apps (ease of use, usefulness, personalization), demographics | Not specified | (Dias et al., 2022) |
| 47 | Service-bots | Improved service quality, utility and hedonic value | Need for human interaction | Attitude towards robotics (service quality, ease of use, usefulness, hedonic value), emotional aspects, demographics | Coffee | (Yoo et al., 2022) |
| 48 | Big Data, online reviews analytics, ML | Larger sample size | Difficulties in data handling, limited accuracy in capturing some variables of consumer behavior | Perceived product and service quality, price perceptions | Not specified | (B. Nguyen et al., 2021) |
| 49 | Big Data | Ability for conduction of spatial analysis | Not reported | Healthiness, eco-friendliness, WTP for organic products, demographics | Organic products | (S. Han & Lee, 2022) |
| 50 | SMA | Large-scale & updated data, eWOM | Limited accuracy in the automated interpretation of some linguistic cues, potential bias | Product quality, healthiness | Not specified | (Jung et al., 2021) |
| 51 | ML | Market segmentation, analysis of complex data | Difficulties in data handling | Product quality (eco-friendliness, healthiness), trust in organic claims, price, demographics | Organic products | (Tohidi et al., 2023) |
| 52 | Video recording | Tracking of eating behavior | Time-consuming & challenging data interpretation, need for tech. expertness, high investment cost | Perceived product quality, demographics | Dairy | (Varela et al., 2021) |
| 53 | ML | Market segmentation, accuracy, large-scale data handling | Overfitting risk, difficulties in data interpretation, requirements of large datasets | Perceived product quality, demographics | Meat | (Luaces et al., 2015) |
| 54 | ΑΙ | Predictive ability | Privacy & security concerns | Demand, price, sustainability, food security | Cereals | (Trollman et al., 2023) |
| 55 | SMA, ML | Updated data, eWOM | Difficulties in data interpretation | Service & product quality, brand, subjective norms, price | Not specified | (Singh R. & Verma H., 2020) |
| 56 | Personalized recommendation system, ML | Personalized experience | Need for human interaction, technological limitations | Perceived product quality & price | Dairy, cereals, snacks, juice, beer | (X. Li et al., 2018) |
| 57 | Service-bots | Improved service quality, hedonic value | Need for human interaction, technological limitations | Attitude towards robotics (hedonic value, ease of use, usefulness, service quality, need for human interaction, technology acceptance), demographics | Not specified | (Santiago et al., 2024) |
| 58 | Big Data, online reviews analytics, ML | Analysis of large-scale & unstructured data | Limited accuracy in the automated interpretation of some linguistic cues, technological complexity | Service & product quality, healthiness, price | Not specified | (Gu et al., 2023) |
| 59 | VR | Simulation of consumer behavior, accuracy, better experimental control, adaptability to contexts | High investment cost | Product type, perceived healthiness & price, quantities purchased, money spent, demographics | Dairy products, snacks | (de-Magistris et al., 2022) |
| 60 | Sensory devices (smart bin) | Educational content, objective measurements | Technological limitations, privacy concerns | Environmental awareness, food waste, subjective norms, healthiness, demographics | Not specified | (Jones-Garcia et al., 2022) |
| 61 | VR, fMRI | Simulation of consumer behavior (VR), objective measurements (fMRI) | Limited telepresence (VR), high investment cost | Product type, product quality (hedonic value, healthiness), telepresence, demographics | Dairy, cereals, snacks, beverages | (van der Laan et al., 2022) |
| 62 | VR | Simulation of consumer behavior, low research cost, motion-tracking, telepresence adaptability to contexts, better experimental control | Unfamiliarity, high investment cost, need for technological advancement | Product placement, purchasing time, quantities purchased | Not specified | (Ploydanai et al., 2017) |
| 63 | SMA | Cost-effective and quicker research method, larger sample size | Not representative sample distribution, limited accuracy in the automated interpretation of some linguistic cues | Product perceptions (healthiness, price, hedonic value), social influence | Alcoholic beverages | (J. Li & Hu, 2021) |
| 64 | Smart devices with personalized services | Personalization, utility value | Privacy & security issues | Product perceptions (healthiness, product type, quality & variety, price), users’ experience (personalization, service quality, usefulness, ease of use), cultural influence, demographics | Snacks | (Vehmas et al., 2019) |
| 65 | VR, EEG | Simulation of consumer behavior, adaptability to contexts (e.g. product placement testing), motion-tracking (VR), objective measurements (EEG) | Users’ unfamiliarity (VR), technological limitations | Product type, eco-friendliness, healthiness, price, quantities purchased, purchasing time, emotional responses, demographics | Fruits, vegetables, dairy, seafood, meat, cereals | (Biercewicz et al., 2022) |
| 66 | ML | Predictive accuracy, analysis of complex data | Not reported | Product perceptions (quality, hedonic value), demographics, emotional & cognitive factors | Meat | (B. Wang et al., 2022) |
| 67 | ML | Accuracy, adaptability to research contexts, large-scale data analysis, market segmentation, personalization | Technological complexity | Perceptions on product attributes (quality, price, product type, brand), impact of promotion, quantities purchased, money spent, purchase frequency, demographics | Fruits, vegetables, cereals | (Donnelly et al., 2021) |
| 68 | Big Data | Analysis of complex, large-scale data | Not reported | Product perceptions (quality, healthiness, price, hedonic & utility value, eco-friendliness) | Not specified | (Jo & Lee, 2021) |
| 69 | ML | Predictive ability, large-scale & complex data handling | High computational cost, need for tech expertness, limited accuracy in the interpretation of some examined variables | GMO-product perceptions (price, quality, risks, healthiness, eco-friendliness, trust), WTP, cognitive aspects, demographics | Seafood | (Weir & Sproul, 2019) |
| 70 | Transactional analytics, ML | Access to larger datasets, market segmentation, predictive accuracy (ML) | Limited accuracy in the interpretation of some variables, requirement of large datasets | Product type, price, buying metrics (money spent, quantities purchased), demographics, promotion | Organic fruits | (Nelson et al., 2019) |
| 71 | ML | Predictive ability, large-scale data analysis | Not reported | Attitude towards grocery apps (usefulness, ease of use, hedonic value), social influence, impact of Covid-19 | Not specified | (Gumasing et al., 2023) |
| 72 | AI facial recognition, ML | Facial tracking (by software supported using social media), predictive accuracy (ML) | Potential bias in data interpretation | Emotional responses, perceived hedonic & utility value of food products | Dairy, nut butters | (Tzafilkou et al., 2023) |
| 73 | VR | Simulation of consumer behavior, low research cost, enhanced hedonic value, telepresence | Not reported | Users’ experience (telepresence, hedonic value) | Not specified | (S. L. Han et al., 2023) |
| 74 | ML | Predictive accuracy, analysis of large-scale & complex data | Challenging data interpretation | Product perceptions (healthiness, brand, eco-friendliness, price), WTP, demographics | Organic products | (Shen et al., 2021) |
| 75 | ML | Market segmentation, convenient data analysis | Not reported | Price perceptions, environmental & health awareness, trust in organic claims, perceived product & service quality, demographics | Organic products | (Boccia & Tohidi, 2024) |
| 76 | VR, sensory technologies | Enhanced hedonic value, telepresence | Users’ unfamiliarity, technological limitations | Users’ experience (ease of use, telepresence, hedonic value), demographics | Dairy | (Peruzzini et al., 2023) |
| 77 | SMA | Larger sample size, low research cost | Not representative sample distribution | Product perceptions (healthiness, eco-friendliness, quality, hedonic value), impact of promotion | Fruits, vegetables, dairy, snacks | (Fatemi et al., 2023) |
| 78 | Big Data, online reviews analytics, ML | Real-time data, larger sample size | Data complexity & challenging data analysis | Brand, perceived product & service quality (ease of use, usefulness) | Foodservice products | (Choi et al., 2024) |
| 79 | ML | Adaptability to research contexts | Not reported | Product perceptions (hedonic value, eco-friendliness, quality, healthiness), social influence, demographics | Organic, alcoholic beverages | (Sukumaran & Majhi, 2024a) |
| 80 | VR | Adaptability to contexts, better control of examined variables, users’ hedonic value | Technological limitations, difficulties in technological use & limited behavioral control | Perceived product quality & price, users’ experience (ease of use, behavioral control, hedonic value), demographics | Snacks | (Zulkarnain et al., 2024a) |
| 81 | VR | Adaptability to contexts, personalization, telepresence | Need for technological advancement, high cost | Users’ experience (telepresence, hedonic value), buying behavior, demographics | Cereals, snacks | (Frank et al., 2024) |
| 82 | Sensory technologies, AI | Market segmentation, personalized services (ML) | Requirement of large datasets (ML), security & privacy issues, need for tech advancement | Buying behavior, personalization | Seafood | (M. Zhang, 2023) |
| 83 | Big Data, SMA | Objectivity, analysis of large-scale & complex data | Challenging data handling, need for technological expertness | Attitude towards food delivery (product & service quality, price, eco-friendliness, utility & hedonic value, Covid-19 impact, healthiness) | Not specified | (Jang et al., 2022b) |
| 84 | ML | Market segmentation, personalization, predictive ability, quicker research method | Need for technological advancement | Product type, perceived product & service quality, price perceptions, environmental & health awareness, frequency of purchases, demographics | Fruits, vegetables, dairy, cereals | (Liashenko et al., n.d.) |
| 85 | Big Data: transactional analytics, ML | Real-time data, market segmentation, personalization, accuracy | Challenging large-scale data handling, high computational cost, privacy - security concerns | Product type, money spent, quantities purchased, healthiness, demographics | Snacks, beverages, cereals | (Izang et al., 2019) |
| 86 | Online reviews analytics | Updated data | Limited accuracy in the automated interpretation of some linguistic cues, not deep understanding of some variables | Cognitive & emotional drivers, consumers’ loyalty, perceived service quality, price, impact of Covid-19 | Not specified | (Kuikka et al., 2024) |
| 87 | Big Data: transactional analytics, ML | Large-scale and updated data, accuracy, objectivity, market segmentation | Challenging data interpretation, overfitting risk, high computational cost | Product type & placement, buying metrics (money spent, quantities purchased, purchasing frequency), price, demographics | Not specified | (Alawadh & Barnawi, 2024) |
| 88 | VR | Simulation of consumer behavior, telepresence, research accuracy | Reduced sensory immersion, need for technological advancement | Product perceptions (hedonic value, quality), demographics | Snacks | (Alba-Martínez et al., 2024) |
| 89 | VR | Simulation of consumer behavior, telepresence & hedonic value | Reduced sensory immersion, limited accuracy in replicating some variables (e.g. purchasing time) | Users’ experience (hedonic value, telepresence), buying behavior (e.g. purchasing time), demographics | Sweet snacks | (Yang et al., 2024) |
| 90 | VR, sensory technologies | Simulation of consumer behavior, cost-effective & quicker research method, telepresence (VR), objective measurements (EEG), ease of use | Not exact replication of physical store, users’ unfamiliarity | Users’ experience (ease of use, behavioral control, telepresence), purchasing time, demographics | Not specified | (Woodall & Hollis, 2024) |
| 91 | Big Data | Personalized marketing: improved service quality | Challenging data analysis | Attitude towards e-shopping (hedonic value, service quality, eco-friendliness, utility value), impact of Covid-19, demographics | Not specified | (Shyu et al., 2023) |
| 92 | Big data, SMA | Larger sample size, objectivity in research | Not reported | Hedonic value of dining out, perceived product & service quality, impact of Covid-19 | Not specified | (Jang et al., 2022a) |
| 93 | ML | Predictive accuracy, market segmentation | Challenging data handling & interpretation | Healthiness, buying behavior, demographics | Organic products | (Jajić et al., 2022) |
| 94 | Smart device with personalized services | Personalization, enhanced utility value | Need for advancement | Product perceptions (quality, variety, hedonic value, healthiness, price), WTP, attitude towards tech application (usefulness, ease of use, service quality), purchasing time, demographics | Cereals, dairy | (Vehmas et al., 2020) |
| 95 | AI driven technology | Objective measurements & data collection, improved service quality, sustainability | Privacy & security issues, technological limitations | Attitude towards tech applications (utility value, service quality, eco-friendliness, subjective norms, privacy & security issues), demographics | Not specified | (Nunkoo et al., 2024) |
| 96 | ML | Objectivity, market segmentation, large-scale data handling | Challenges in data interpretation (LDA) | External influence, food safety, expenditure, demographics | Not specified | (T. Li et al., 2024) |
| 97 | AI | Quicker research method, accuracy, updated data and large-scale data handling | Technological limitations, potential bias | Healthiness, product quality | Snacks, beverages, cereals, dairy | (Kim et al., 2024) |
| 98 | Big Data | Large-scale data, predictive ability | High cost, security concerns, challenging data analysis | Price, product quality, demand, quantity purchased | Fresh products | (Dey et al., 2024) |
| 99 | ML | Objectivity | Not deep understanding of some examined variables | Product perceptions (price, healthiness, quality, brand, eco-friendliness), trust in organic claims, demographics | Organic & sustainable food products | (Çakmakçi et al., 2024) |
| 100 | Big Data, web scraping | Large-scale data, real-time pricing, predictive ability, low-cost research method | Technological complexity | Product type, price, quantities purchased | Cereals, beverages, legumes | (Muñoz-Villamizar et al., 2024) |
| 101 | ML | Market segmentation, large-scale data handling, research accuracy | Not reported | Product perceptions (type, quality, hedonic value, animal welfare, healthiness, price), WTP, demographics | Eggs | (Arno et al., 2023) |
| 102 | Food apps | eWOM, data with various insights | Potential bias | Digital feedback intention (social influence, usefulness, ease of use, privacy & security concerns), demographics | Not specified | (Haruyama & Hidaka, 2024) |
| 103 | Big Data | Low-cost & quicker research method, real-time & large-scale data | Not representative sample distribution, not deep understanding of examined variables | Product perceptions | EU quality-certified products (PDO/PGI/TSG) | (Glogovețan et al., 2023) |
| 104 | ML | Personalization, predictive accuracy | Not reported | Product type, price impact, quantities purchased, demographics | Alcoholic beverages | (Zhu et al., 2023) |
| 105 | ML | Quick & easy data interpretation (DT) | Limited accuracy (Naive Bayes) | WTP & perceptions on local products (quality, healthiness, eco-friendliness, trust), demographics | Walnuts | (Çukur et al., 2022) |
| 106 | Big data | Large-scale & updated data | Privacy & security concerns (big data), challenging data interpretation (ML) | Healthiness, eco-friendliness, buying behavior | Not specified | (Timotijevic et al., 2022) |
| 107 | Gamified app | Cost-effective & quicker research method, real-time data, market segmentation, personalization, hedonic value, enhancing of product development before releasing | Potential bias/ misinformation, technological complexity | Perceived product quality | Beverages | (Jreissat & Makatsoris, 2022) |
| 108 | Big Data | Large-scale & updated data | Limited accuracy in the interpretation of some variables | Product type, price, demand, impact of Covid-19 | Not specified | (Gadzalo et al., 2020) |
| 109 | Transactional analytics, ML | Large-scale & objective data (transactional records), quicker research method, predictive accuracy (ML) | Not reported | Product type, money spent, quantities purchased, demographics | Processed food (noodles) | (Lahindah & Diryana Sudirman, 2023) |
| 110 | VR | Eye-tracking, simulation of consumer behavior, better experimental control, objective outcomes, low-research cost, telepresence | Not reported | Product type, purchasing time, product perceptions (hedonic value, price, animal welfare), telepresence, demographics | Meat, poultry, plant-based meat | (Xu et al., 2023) |
| 111 | Transactional analytics, ML | Larger sample size, quicker research method, accuracy | Challenges in data interpretation, potential selection bias | Product type, impact of promotional stimuli, buying metrics (e.g. money spent), demographics | Multiple food & non-food products | (Langen & Huber, 2023) |
| 112 | ML | Predictive ability | Requirement of large datasets | Product perceptions (healthiness, quality, price, hedonic value), purchase frequency, impact of Covid-19, demographics | Poultry | (Chiras et al., 2023) |
| 113 | Big Data: online reviews analytics, recommendation system, ML | Personalization, large-scale data, accuracy, improved service quality | Unstructured data, limited accuracy in the automated interpretation of some linguistic cues, technological complexity, privacy issues | Product perceptions - green consumption behavior | Sustainable & organic food products | (Geng et al., 2023) |
| 114 | Big data: transactional analytics | Larger sample size, quicker method, objective data | Not deep understanding of some variables | Product type, purchase frequency, quantities purchased, consumption behavior, demographics | Vegetables, legumes, cereals, snacks | (Vepsäläinen et al., 2022) |
| 115 | VR | Simulation of consumer behavior, better control of examined variables, telepresence, accuracy | Reduced sensory immersion, limited accuracy in replicating some variables, tech limitations | Product perceptions (type, quality, hedonic value, healthiness, eco-friendliness), users’ experience (telepresence, ease of use), demographics | Cereals, vegetables, seafood, eggs, dairy | (Arrazat et al., 2023) |
| 116 | ML | Low-cost & quicker method, predictive accuracy, large-scale data handling, adaptability to research contexts | Need for technological expertness, not deep understanding of examined variables | Perceptions on product packaging (eco-friendliness, utility value) | Beverages, snacks, cereals | (Parcesepe et al., 2023) |
| 117 | Sensory technologies, ML | Predictive ability, quicker research method (ML), objective measurements | Possible misclassification | Emotional responses, product perceptions (quality and hedonic value), demographics, | Dairy and plant-based products | (Gupta et al., 2022) |
| 118 | Transactional analytics | Market segmentation, objective data, quicker research method | Privacy concerns | Buying metrics (product type and quantities purchased, purchase frequency, money spent), healthiness, demographics | Meat, seafood, dairy, cereals | (Morris et al., 2020) |
| 119 | Personalized services | Personalization, market segmentation, enhancing of healthiness | Technological complexity | Healthiness, hedonic value, impact of personalized promotion, demographics | Not specified | (M. T. T. Nguyen et al., 2021) |
| 120 | Big Data, online reviews analytics, ML | Real-time & unbiased data | Unavailability of demographics, limited accuracy in the automated interpretation of some linguistic cues | Product type, perceived product & service quality, (hedonic value, healthiness), price perceptions, impact of Covid-19 | Not specified | (C. Zhang et al., 2021) |
| 121 | Transactional analytics | Objective data, market segmentation | Not representative sample distribution | Product type, buying behavior, demographics | Animal-based & processed products, fruits, vegetables | (Jiyoung & Heedae, 2020) |
| 122 | Transactional analytics | Objectivity, cost-effective research | Unavailability of demographics due to privacy issues, not representative sample distribution, not deep understanding of some variables | Product type, healthiness | Protein sources | (Green et al., 2020) |
| 123 | VR | Simulation of consumer behavior, controlled setting | Limited accuracy in the replication of all attributes of a physical store, users’ unfamiliarity and drop-out rate | Product type, healthiness, impact of promotion, purchasing time, telepresence, demographics | Snacks, dairy products, beverages, cereals | (Blom et al., 2021) |
| 124 | Service-bots | Improved service quality, hedonic value | Technological limitations, users’ unfamiliarity | Attitude towards robotics (trust, usefulness, hedonic value, subjective norms, service quality), demographics | Not specified | (Lee et al., 2021) |
| 125 | Big data: transactional analytics | Cost-effective and quicker research method, objective & large-scale data | Privacy & security issues, not deep understanding of all examined variables, not representative sample distribution | Healthiness, buying metrics (product type and quantities bought, money spent), demographics | Not specified | (Clark et al., 2021) |
| 126 | Big data: transactional analytics, ML | Predictive ability, updated and large-scale data | Potential bias & limited accuracy in the interpretation of some data, privacy & security issues | Buying metrics (product type and quantities purchased), healthiness | Not specified | (Miliou et al., 2021) |
| 127 | VR | Enhanced telepresence | Technological limitations (accessibility) | Users’ experience (telepresence, ease of use), perceived price & product quality, demographics | Fruits | (Park et al., 2021) |
| 128 | VR | Simulation of consumer behavior, adaptability to research contexts, better control of examined variables | Limited accuracy in the simulation of some aspects of buying behavior & physical environment | Preferred product type, healthiness, purchasing time, telepresence, demographics | Snacks, cereals | (Blitstein et al., 2020) |
| 129 | VR | Simulation of consumer behavior, better control of examined variables, objective measurements | Limited accuracy in the simulation of some aspects of buying behavior, potential bias | Impact of price, buying metrics (preferred product type, expenditure, quantity bought), healthiness, demographics | Fruits, vegetables, dairy, snacks | (Hoenink et al., 2020) |
| 130 | Other | Real-time data, better control of examined variables, educational content, personalization | Not deep understanding of behavioral factors | Healthiness, demographics | Not specified | (Shin et al., 2020) |
| 131 | MR | Enhancement of users’ hedonic value, better control of examined variables, simulation of consumer behavior | Potential bias/ misinformation | Users’ experience (hedonic value & sentimental dimensions), perceived product quality, demographics | Sweet snacks | (Low et al., 2021) |
| 132 | Apps with personalized services | Personalization, educational content | Privacy & security concerns | Healthiness, perceptions on personalization, privacy concerns, demographics | Not specified | (Reinders et al., 2020) |
| 133 | Apps with personalized services | Personalization, improved service quality, educational content | Privacy & security concerns | Perceptions & WTP for personalized services, healthiness, demographics | Not specified | (Pérez-Troncoso et al., 2021) |
| 134 | Personalized services | Personalization, improved service quality, educational content | Technological limitations | Perceived product quality, attitude towards personalization (utility value, benefits, risks) | Not specified | (Calegari et al., 2018) |
| 135 | Big Data, SMA | Updated data with deep insights, eWOM | Potential misinformation / bias, not representative sample distribution | Product type, perceptions on service & product quality (healthiness, hedonic value) | Not specified | (Ariyasriwatana & Quiroga, 2016) |
| 136 | Big Data: transactional analytics, ML | Large-scale and updated data, predictive accuracy | Complex data, challenging data interpretation, not deep understanding of behavioral aspects | Perceived product & service quality, impact of price and discounts, social influence | Not specified | (Mu, 2019) |
| 137 | Big Data, ML | Updated and large-scale data, cost-effective and quicker research method | Limited accuracy in sentimental data interpretation, need for technological expertness, privacy issues | Product perceptions (quality, healthiness, eco-friendliness, price, hedonic, utility & social value), demographics | Not specified | (T. Y. Chen et al., 2016) |
| 138 | Big data: transactional analytics | Not reported | Not reported | Product perceptions (healthiness, quality, eco-friendliness, price), demographics | Organic & sustainable food products | (Luo et al., 2015) |
| 139 | Big data, transactional analytics | Objectivity, large-scale data | Not reported | Healthiness, buying metrics (product type and quantities purchased), demographics | Dairy, meat, beverages | (Grummon & Taillie, 2017) |
| 140 | Big data, SMA | Larger sample size, real-time & unbiased data | Unstructured data and challenging data analysis, privacy issues, not representative sample distribution | Product perceptions (quality, healthiness, eco-friendliness, price, promotion) | Meat | (Mishra et al., 2017) |
| 141 | VR | Objectivity, simulation of consumer behavior, better experimental control | Limited accuracy in the replication of some examined variables | Product perceptions (healthiness, price, quality), buying metrics (money spent, product type and quantities bought), demographics | Not specified | (Payne Riches et al., 2019) |
| 142 | VR | Cost-effective and quicker method, simulation of consumer behavior, better experimental control, research accuracy, enhanced telepresence | Users’ drop-out rate, technological limitations, limited accuracy in the simulation of some variables | Buying metrics (product type and quantities purchased, money spent), telepresence, demographics | Cereals, snacks, beverages, fruits | (Waterlander et al., 2015) |
| 143 | Big data: transactional analytics | Cost-effective method, large-scale data | Not deep understanding of some behavioral factors | Buying metrics (purchase frequencies, money spent, product type purchased), impact of price, discounts, variety and brand, consumers’ age & gender | Cereals, snacks, beverages, dairy | (Tian et al., 2018) |
| 144 | Big data, ML, personalized services | Large-scale data, predictive accuracy, personalization, market segmentation | Challenging data analysis & interpretation, high computational & investment cost | Product perceptions, price, buying behavior | Not specified | (Kanavos et al., 2018) |
| 145 | VR, EEG | Objective measurements (EEG), simulation of consumer behavior (VR) | Time-consuming (EEG), high cost | Impact of product type and placement, buying metrics (purchasing time) | Fruits, vegetables, dairy, seafood, meat, cereals | (Biercewicz et al., 2024) |
| 146 | ML | Market segmentation, large-scale data handling, predictive ability | Not reported | Perceived product quality & price, demographics, healthiness, eco-friendliness | Cereals, vegetables, beverages | (Burke et al., 2025) |
| 147 | Big Data: Online reviews analytics | Large-scale data & updated data, quicker research method, eWOM | Potential bias, limited accuracy in the interpretation of some linguistic cues (NLP) | Perceived service quality (utility & hedonic value), price, social influence, impact of Covid-19 | Not specified | (Demydyuk et al., 2024) |
| 148 | Big Data, SMA | Large-scale & updated data, geolocation data available | Challenging data handling, not representative sample distribution, limited accuracy in interpreting some linguistic cues (NLP) | Product perceptions (eco-friendliness, healthiness, quality), price, impact of Covid-19 | Meat | (Fisk et al., 2024) |
| 149 | Video recording, ML | Quicker research method, analysis of large-scale data (ML), deep insights | Challenging interpretation of some linguistic cues, technological limitations | Sustainability & eco-friendliness, subjective norms | Not specified | (Gul et al., 2024) |
| 150 | ML | Market segmentation, predictive ability | Not reported | Impact of promotion, eco-friendliness, cultural influence, demographics | Meat, oil, honey | (Huo et al., 2024) |
| 151 | SMA, ML | Large-scale data, predictive accuracy | Overfitting risk, not deep understanding of examined variables & behavioral factors | Brand perception, perceived service & product quality, healthiness, impact of Covid-19 | Not specified | (Ju, 2024) |
| 152 | VR | Simulation of consumer behavior, adaptability to research contexts, accuracy, telepresence | Unfamiliarity, technological limitations | Product perceptions (quality, hedonic value, brand), user experience (telepresence, hedonic value), demographics | Meat | (Man et al., 2024) |
| 153 | ML | Predictive accuracy, analysis of complex data | Challenging data interpretation, overfitting risk, technological complexity, requirement of large datasets | Product perceptions (quality & hedonic value), demographics | Fruits | (Natsume & Okamoto, 2024) |
| 154 | Personalized services (AI) | Personalization, enhanced utility value & service quality | Technological anxiety | User experience (service quality, perceived risks, behavioral control, utility value), demographics | Beverages | (Rohden & Espartel, 2024) |
| 155 | Transactional analytics, ML | Market segmentation, large-scale data, research accuracy | High computational cost, challenging data handling & interpretation | Purchase frequency, money spent, impact of discounts, eco-friendliness, demographics | Not specified | (Sakaline & Buics, 2024) |
| 156 | ML | Large-scale data, market segmentation, predictive ability | Overfitting risk | Product perceptions (quality, hedonic value, healthiness, eco-friendliness, price), subjective norms, demographics | Organic, alcoholic beverages | (Sukumaran & Majhi, 2024b) |
| 157 | Online reviews analytics, ML | Larger sample size, accuracy, updated data | Challenging data interpretation, technological complexity | Perceived price fairness, product & service quality | Not specified | (Tan et al., 2025) |
| 158 | AI- and AR-driven nutrition app with personalized services | Utility value, users’ engagement, educational content, personalization | Technological limitations, need for advancement | User experience (usefulness, ease of use, technology acceptance), healthiness, demographics | Not specified | (Campos et al., 2024) |
| 159 | VR | Telepresence, better experimental control, product testing before releasing | Technological limitations, need for advancement | Perceived product quality, user experience (telepresence, ease of use, behavioral control, hedonic value), demographics | Snacks | (Zulkarnain et al., 2024b) |

Table 2: Information on the methodology used in the reviewed studies

| N | Type of technology applied/ examined | Theoretical frameworks used | Sample size | Age group of participants | Geographical level of data collection | Methodological approach | Data analysis techniques | Comparison / combination of tech-based with traditional research methods | Reference |
| --- | --- | --- | --- | --- | --- | --- | --- | --- | --- |
| 1 | SMA | N/A | 500 | Not reported | National | Quantitative | Correlation Analysis, Frequency Analysis, SNA | N/A | (Culotta & Cutler, 2016) |
| 2 | VR | N/A | 100 | 18 - 74 | Local | Quantitative | ANOVA, PCA | ✓ | (van Herpen et al., 2016) |
| 3 | VR | N/A | 68 | Mean age: 24 | Local | Quantitative | t-test | N/A | (Siegrist et al., 2019) |
| 4 | Big Data, SMA | N/A | Not reported | Not reported | National | Quantitative | Frequency & Sentiment Analysis (NLP-based), Classification Analysis, Association Analysis | N/A | (Mishra & Singh, 2018) |
| 5 | Apps with personalized services | Privacy Calculus, TAM | 348 | 20 - 50+ | National | Quantitative | SEM, CFA | N/A | (Kang & Namkung, 2019) |
| 6 | VR | N/A | 41 | 23 - 61 | Local | Qualitative | fsQFA | N/A | (Bigné et al., 2016) |
| 7 | VR | TAM, Flow Theory | 120 | 20 – 40+ | National | Quantitative | SEM, CFA | N/A | (S. L. Han et al., 2020) |
| 8 | VR | N/A | 111 | 20 - 29 | Local | Mixed | Thematic Analysis, ANOVA | ✓ | (Schnack et al., 2019) |
| 9 | Apps with personalized services | TAM,  SERVQUAL | 494 | 15 - 65+ | National | Quantitative | PLS-SEM | N/A | (Su, Nguyen, et al., 2022) |
| 10 | VR | Cue Utilization Theory | 142 | 19 - 23 | Local | Quantitative | CFA, PLS-SEM, ANOVA, t-test | N/A | (Lombart et al., 2019) |
| 11 | VR | TRA | 192 | 18 - 22 | Local | Quantitative | PLS-SEM, ANOVA, t-test | ✓ | (Lombart et al., 2020) |
| 12 | Food apps | UTAUT2 | 443 | 18 - 51+ | National | Quantitative | CFA, Mediation Analysis | N/A | (Habib & Hamadneh, 2021) |
| 13 | VR | N/A | 63 | 20 - 57 | Local | Quantitative | ANOVA | ✓ | (Andersen et al., 2019) |
| 14 | Nutrition apps with personalized services | N/A | 18 | Mean age: 54 | Local | Qualitative | Thematic Analysis | N/A | (Peng et al., 2016) |
| 15 | Big Data: online reviews analytics | Ν/Α | 686000* | Not reported | National | Quantitative | Sentiment Analysis, Time-series Analysis | N/A | (Brandtner et al., 2021) |
| 16 | Service-bots | TAM | 317 | 20 - 60+ | National | Quantitative | CFA, SEM, t-test | N/A | (Sung & Jeon, 2020) |
| 17 | Service-bots | CASA Theory | 1370 | 18 - 87 | National | Mixed | SEM, CFA, ANOVA, Thematic Analysis | N/A | (Song & Kim, 2022) |
| 18 | ML | TPB, IBT, GFT, TIB | 1003 | 36 - 45 | National | Quantitative | Correlation Analysis, PCA, Cluster Analysis (DBSCAN), Classification Analysis (SVM, LR, DT) | N/A | (Taghikhah et al., 2021) |
| 19 | VR | Service Performance Model | 50 | 20 - 30 | Local | Quantitative | Descriptive statistics only | N/A | (Violante et al., 2019) |
| 20 | Online reviews analytics, ML | SERVQUAL | 47548* | Not reported | National | Quantitative | Sentiment analysis**,** TF-IDF, LDA | N/A | (S. Chen et al., 2023) |
| 21 | VR | N/A | 256 | 18 - 22+ | Local | Quantitative | t-test | ✓ | (Fang et al., 2021) |
| 22 | VR | N/A | 153 | 13 - 77 | Local | Quantitative | t-test | N/A | (Schnack et al., 2020) |
| 23 | Big Data: SMA, ML | N/A | 4461* | Not reported | Local | Quantitative | Cluster analysis (SOM), LDA, Classification Analysis (RT-CART) | N/A | (Nilashi et al., 2021) |
| 24 | ML, EEG | N/A | 203 | 19 - 41 | Local | Quantitative | PCA, ANOVA, Mediation Analysis, Correlation analysis, t-test, Classification Analysis (SVM, DT) | ✓ | (Hakim et al., 2021) |
| 25 | SMA | Gronroos’s Service Quality Model | 533* (online reviews), 494 (questionnaires) | 18 – 65+ | National | Mixed | Thematic analysis, PLS-SEM, PCA | ✓ | (Su, Nguyen-Phuoc, et al., 2022) |
| 26 | Apps with personalized services | Privacy Calculus | 8136 | 18 - 65 | Cross-national | Quantitative | CFA, SEM | N/A | (Berezowska et al., 2015) |
| 27 | SMA, ML | Situational Crisis Communication Theory | 161921 * | Not reported | National | Quantitative | LDA, Time-series Analysis, Sentiment Analysis, Frequency Analysis | N/A | (X. Li et al., 2023) |
| 28 | Big Data: SMA, ML | N/A | 43724 * | Not reported | Global | Quantitative | Frequency & Sentiment Analysis (NLP-based), LDA | N/A | (Singh & Glińska-Neweś, 2022) |
| 29 | Big Data: SMA, ML | N/A | 13635 * | Not reported | Not reported | Quantitative | Frequency & Sentiment analysis (NLP-based), LDA | N/A | (Brzustewicz & Singh, 2021) |
| 30 | VR | N/A | 32 | 18 - 38 | Local | Quantitative | Correlation Analysis, ANOVA | N/A | (Q. J. Wang et al., 2020) |
| 31 | Service-bots | N/A | 22 | 21 - 40+ | Local | Qualitative | Thematic Analysis, Frequency Analysis | N/A | (Rasheed et al., 2023) |
| 32 | VR | N/A | 98 | 19 - 71 | National | Quantitative | Correlation & Classification Analysis | ✓ | (Xu et al., 2021) |
| 33 | VR | N/A | 50 | 18 - 50 | Local | Quantitative | ANOVA, PCA, Cluster Analysis | N/A | (Torrico et al., 2021) |
| 34 | Big data: SMA, ML, TL | N/A | 41782 * | Not reported | National | Quantitative | Sentiment Analysis (NLP-based), Classification Analysis (Bayesian classifier, SVM), TF-IDF, LDA, TL (BERT) | N/A | (Y. Chen & Zhang, 2022) |
| 35 | Big Data: SMA | SERVQUAL | 410309* | Not reported | National | Quantitative | Frequency & Sentiment analysis (NLP-based) | N/A | (He et al., 2018) |
| 36 | Transactional analytics, ML | N/A | 2000 | Not reported | Cross-national | Quantitative | 2way-MF, MFDL | N/A | (S. S. Chen et al., 2021) |
| 37 | Chatbots, ML: Transfer learning | N/A | 30 | 20 - 45 | Local | Quantitative | Classification Analysis (CART, DT) | N/A | (Chiu & Chuang, 2021) |
| 38 | Big Data: SMA, ML | N/A | 7014 * | Not reported | Global | Quantitative | Cluster Analysis (DBSCAN), Sentiment Analysis | N/A | (Pindado & Barrena, 2021) |
| 39 | Big Data, online reviews analytics | N/A | 4531353*, 8002 questionnaires, 40 interviews | Not reported | National | Mixed | TF-IDF, Classification Analysis, Semantic Analysis, Correlation Analysis, Regression Analysis, ANOVA, SNA | ✓ | (B. Wang et al., 2023) |
| 40 | VR | N/A | 334 | 18 - 70 | Local | Quantitative | ANOVA | N/A | (Goedegebure et al., 2020) |
| 41 | Big Data, ML | N/A | ≈323000 * | Not reported | National | Quantitative | Regression Analysis (RF), Time-series analysis, Correlation Analysis | ✓ | (Ryu et al., 2020) |
| 42 | ML | N/A | 700 | 18 - 50+ | National | Quantitative | Classification Analysis (DT) | Ν/A | (Vindigni et al., 2022) |
| 43 | Big Data, SMA | N/A | >14.000.000* | Not reported | National | Quantitative | Sentiment analysis (NLP-based) | N/A | (Widmar et al., 2020) |
| 44 | Online food apps | Behavioral Perspective Model | 294 | Not reported | Local | Quantitative | Choice-based conjoint analysis | N/A | (Sigurdsson et al., 2017) |
| 45 | ML | N/A | 715 | Not reported | Cross-national | Quantitative | Cluster analysis (GMM), Classification analysis (MLP) | N/A | (Salamzadeh et al., 2022) |
| 46 | AI-driven gamified nutrition app with personalized services | TAM | 85 | 25 - 44 | Cross-national | Quantitative | CFA, Regression Analysis, Mediation Analysis | N/A | (Dias et al., 2022) |
| 47 | Service-bots | N/A | 300 | 20 - 60+ | Local | Quantitative | EFA, PCA, CFA, SEM | N/A | (Yoo et al., 2022) |
| 48 | Big Data, online reviews analytics, ML | N/A | 236867* | Not reported | National | Quantitative | Time-series Analysis, Sentiment Analysis, Classification Analysis (DT, Bayesian classifier, SMV, RT) | N/A | (B. Nguyen et al., 2021) |
| 49 | Big Data | N/A | 3330 | 18 -74 | National | Quantitative | Correlation analysis, Ordered Probit Model | N/A | (S. Han & Lee, 2022) |
| 50 | SMA | N/A | 2192450* | Not reported | National | Quantitative | Frequency & Sentiment Analysis (NLP-based), Time-series analysis, Correlation Analysis | N/A | (Jung et al., 2021) |
| 51 | ML | SOR model | 400 | Not reported | Local | Quantitative | Cluster analysis | N/A | (Tohidi et al., 2023) |
| 52 | Video recording | N/A | 103 | 18 - 24 | Local | Quantitative | PCA, ANOVA | N/A | (Varela et al., 2021) |
| 53 | ML | N/A | 392 | Not reported | Local | Quantitative | Cluster Analysis, Classification Analysis (SVM) | N/A | (Luaces et al., 2015) |
| 54 | ΑΙ | N/A | N/A | N/A | N/A | Qualitative (conceptual) | N/A | N/A | (Trollman et al., 2023) |
| 55 | SMA, ML | N/A | 4000* | Not reported | National | Quantitative | Sentiment Analysis, Classification Analysis (RF), Matrix Factorization | N/A | (Singh R. & Verma H., 2020) |
| 56 | Personalized recommendation system, ML | N/A | 37 | 20 - 60 | Local | Quantitative | Matrix Factorization (MARMTF) | N/A | (X. Li et al., 2018) |
| 57 | Service-bots | TAM | 293 | 18 - 59 | National | Quantitative | PLS-SEM | N/A | (Santiago et al., 2024) |
| 58 | Big Data, online reviews analytics, ML | N/A | 372997* | Not reported | National | Quantitative | LDA | N/A | (Gu et al., 2023) |
| 59 | VR | N/A | 323 | 18 - 55+ | Local | Quantitative | ANOVA, t-test | ✓ | (de-Magistris et al., 2022) |
| 60 | Sensory devices (smart bin) | N/A | 31 | Not reported | Local | Mixed | Frequency Analysis, Thematic Analysis | ✓ | (Jones-Garcia et al., 2022) |
| 61 | VR, fMRI | N/A | 56 | 18 - 26 | Local | Quantitative | ANOVA, t-test, Correlation Analysis | N/A | (van der Laan et al., 2022) |
| 62 | VR | N/A | 241 | Not reported | Local | Quantitative | ANOVA | N/A | (Ploydanai et al., 2017) |
| 63 | SMA | N/A | 723 | Mean age: 22 | National | Qualitative | Content Analysis (manual) | N/A | (J. Li & Hu, 2021) |
| 64 | Smart devices with personalized services | N/A | 454 | Mean age (per phase): 35, 41, 42, 52 | Cross-national | Mixed | Thematic Analysis, t-test | N/A | (Vehmas et al., 2019) |
| 65 | VR, EEG | N/A | 34 | Not reported | Local | Quantitative | Correlation Analysis, Sentiment Analysis | N/A | (Biercewicz et al., 2022) |
| 66 | ML | N/A | 169 | 18 - 26 | Local | Mixed | Thematic Analysis, Frequency Analysis, ANOVA, SNA, Classification Analysis (SVM, DT) | Ν/Α | (B. Wang et al., 2022) |
| 67 | ML | N/A | 2068 | Not reported | Local | Quantitative | Matrix Factorization, Bayesian modeling | ✓ | (Donnelly et al., 2021) |
| 68 | Big Data | N/A | 1103 | 18 - 64 | Cross-national | Quantitative | Frequency Analysis, Correlation Analysis | N/A | (Jo & Lee, 2021) |
| 69 | ML | N/A | 1043 | Mean age: 36 | National | Quantitative | Classification Analysis (LASSO, LR) | ✓ | (Weir & Sproul, 2019) |
| 70 | Transactional analytics, ML | N/A | 60000 | Not reported | National | Quantitative | Classification analysis (LASSO, LR) | ✓ | (Nelson et al., 2019) |
| 71 | ML | UTAUT2, PMT | 373 | 18 - 50 | National | Quantitative | Classification analysis (RF), ANOVA | Ν/A | (Gumasing et al., 2023) |
| 72 | AI facial recognition, ML | N/A | 76 | 18 - 50+ | Local | Quantitative | Classification analysis (MLP, RF, DT), Sentiment Analysis | N/A | (Tzafilkou et al., 2023) |
| 73 | VR | SOR model, Flow Theory, Experience Economy Theory | 120 | 20 - 40+ | Local | Quantitative | CFA, SEM, En Analysis | N/A | (S. L. Han et al., 2023) |
| 74 | ML | N/A | 740 | 18 - 65+ | National | Quantitative | Classification Analysis (SVM, RF, LR) | ✓ | (Shen et al., 2021) |
| 75 | ML | SOR model | 400 | Mean age (per segment): 28–55 | Local | Quantitative | Cluster Analysis (SOM), Classification Analysis (DT) | N/A | (Boccia & Tohidi, 2024) |
| 76 | VR, sensory technologies | TPB | 76 | 20 - 60 | Local | Quantitative | Correlation Analysis, t-test | N/A | (Peruzzini et al., 2023) |
| 77 | SMA | N/A | 943779* | Not reported | Global | Quantitative | Classification Analysis, Frequency Analysis | N/A | (Fatemi et al., 2023) |
| 78 | Big Data, online reviews analytics, ML | TAM | 13440*, 343 (questionnaire) | 18 - 65+ | National | Quantitative | LDA, Frequency Analysis, Time-series Analysis, CFA, PLS-SEM | Ν/A | (Choi et al., 2024) |
| 79 | ML | TPB, ΙΒΤ, GFT | 334 | 23 - 45+ | Local | Quantitative | Classification Analysis (DT) | N/A | (Sukumaran & Majhi, 2024a) |
| 80 | VR | N/A | 60 | Not reported | Local | Quantitative | Cluster Analysis, t-test | N/A | (Zulkarnain et al., 2024a) |
| 81 | VR | N/A | 127 | 18 - 70 | Local | Quantitative | ANOVA | N/A | (Frank et al., 2024) |
| 82 | Sensory technologies, AI | N/A | Ν/Α | N/A | Ν/Α | Conceptual quantitative framework | Ν/Α | N/A | (M. Zhang, 2023) |
| 83 | Big Data, SMA | N/A | Not reported | Not reported | National | Quantitative | TF-IDF, CONCOR Analysis, Sentiment analysis (NLP-based) | N/A | (Jang et al., 2022b) |
| 84 | ML | N/A | 141 | Not reported | Local | Quantitative | Cluster Analysis, Classification Analysis (RF), PCA | N/A | (Liashenko et al., n.d.) |
| 85 | Big Data: transactional analytics, ML | TPB | 1112000* | Not reported | Local | Quantitative | Time-series Analysis, Cluster Analysis, Classification Analysis (DT, Bayesian classifier), Frequency Analysis, Correlation Analysis | N/A | (Izang et al., 2019) |
| 86 | Online reviews analytics | N/A | 45603* | Not reported | National | Quantitative | Frequency Analysis, Sentiment Analysis, ANOVA, Correlation Analysis, Regression Analysis | N/A | (Kuikka et al., 2024) |
| 87 | Big Data: transactional analytics, ML | N/A | 29356 | Not reported | National | Quantitative | Cluster Analysis, Classification Analysis (DT, SVM) | N/A | (Alawadh & Barnawi, 2024) |
| 88 | VR | N/A | 103 | Not reported | Local | Quantitative | PCA, ANOVA, Cluster Analysis | N/A | (Alba-Martínez et al., 2024) |
| 89 | VR | N/A | 80 | 18 - 26 | Local | Quantitative | ANOVA | ✓ | (Yang et al., 2024) |
| 90 | VR, sensory technologies | N/A | 31 | 18 - 60 | Local | Quantitative | ANOVA | N/A | (Woodall & Hollis, 2024) |
| 91 | Big Data | N/A | 806 | Not reported | Local | Quantitative | PCA, SEM | N/A | (Shyu et al., 2023) |
| 92 | Big data, SMA | N/A | Not reported | Not reported | National | Quantitative | TF-IDF, SNA, CONCOR Analysis, Sentiment Analysis (NLP-based) | N/A | (Jang et al., 2022a) |
| 93 | ML | Microeconomic Consumer Theory | 252 | 18 – 55+ | National | Quantitative | Classification Analysis (MLP) | N/A | (Jajić et al., 2022) |
| 94 | Smart device with personalized services | N/A | 143 | 15 – 60+ | Local | Mixed | ANOVA, t-test, Thematic Analysis | N/A | (Vehmas et al., 2020) |
| 95 | AI driven technology | Innovation Resistance theory | 1260 | 20 - 50+ | Local | Mixed | Thematic Analysis, PLS-SEM | N/A | (Nunkoo et al., 2024) |
| 96 | ML | N/A | 7835 | Mean age: 51 | National | Quantitative | LDA, Sentiment Analysis (NLP-based), Frequency Analysis, Regression Analysis | N/A | (T. Li et al., 2024) |
| 97 | AI | N/A | N/A (not consumer sample) | N/A | N/A | Quantitative | SNA, Frequency Analysis | N/A | (Kim et al., 2024) |
| 98 | Big Data | N/A | N/A | N/A | N/A | Qualitative (conceptual modeling) | N/A | N/A | (Dey et al., 2024) |
| 99 | ML | N/A | 171 | Mean age: 36 | Local | Quantitative | PCA, Classification Analysis | N/A | (Çakmakçi et al., 2024) |
| 100 | Big Data, web scraping | N/A | N/A (price data, no consumer sample) | N/A | N/A | Quantitative | PCA, Cluster Analysis, Time-series Analysis | N/A | (Muñoz-Villamizar et al., 2024) |
| 101 | ML | N/A | 1415 | Not reported | National | Quantitative | EFA, Cluster Analysis (HCA), Classification Analysis (CDA) | N/A | (Arno et al., 2023) |
| 102 | Online reviews analytics | TAM, TPB | 778 | 18 - 60+ | National | Quantitative | EFA, PLS-SEM, Correlation Analysis | N/A | (Haruyama & Hidaka, 2024) |
| 103 | Big Data | N/A | N/A (Google Trends data, not consumer sample) | N/A | N/A | Quantitative | Frequency Analysis, Time-series Analysis | N/A | (GLOGOVEȚAN et al., 2023) |
| 104 | ML | Utility Theory | 484 | Mean age: 49 | National | Quantitative | Classification Analysis (SVM, RF) | N/A | (Zhu et al., 2023) |
| 105 | ML | N/A | 382 | Mean age: 29 | Local | Quantitative | Classification Analysis (DT, RF, Bayesian classifier) | N/A | (Çukur et al., 2022) |
| 106 | Big data | N/A | N/A | N/A | N/A | Qualitative (conceptual) | N/A | N/A | (Timotijevic et al., 2022) |
| 107 | Gamified app | N/A | 120 | Not reported | Local | Quantitative | PCA, ANOVA, t-test, Cluster Analysis, Correlation Analysis | Ν/A | (Jreissat & Makatsoris, 2022) |
| 108 | Big Data | N/A | N/A (Google Trends Data) | N/A | N/A | Quantitative | Frequency Analysis, Classification Analysis | Ν/A | (Gadzalo et al., 2020) |
| 109 | Transactional analytics, ML | N/A | 474* | Not reported | Local | Quantitative | Classification Analysis (DT, Bayesian classifier) | N/A | (Lahindah & Diryana Sudirman, 2023) |
| 110 | VR | N/A | 124 | 19 - 82 | Local | Quantitative | ANOVA, t-test, Correlation Analysis | N/A | (Xu et al., 2023) |
| 111 | Transactional analytics, ML | N/A | 1582 | 18 - 70+ | Local | Quantitative | t-test, Frequency Analysis, DML, Casual Forests | Ν/A | (Langen & Huber, 2023) |
| 112 | ML | N/A | 689 | 18 - 75+ | Local | Quantitative | Classification Analysis (RF, SVM) | N/A | (Chiras et al., 2023) |
| 113 | Big Data: online reviews analytics, recommendation system, ML | N/A | 9777* | Not reported | National | Quantitative | Frequency Analysis, Sentiment Analysis, Correlation Analysis, Regression Analysis | N/A | (Geng et al., 2023) |
| 114 | Big data: transactional analytics | N/A | 11983 | 18 - 93 | National | Quantitative | Correlation Analysis | ✓ | (Vepsäläinen et al., 2022) |
| 115 | VR | N/A | 132 | 18 - 65 | National | Quantitative | Regression Analysis | N/A | (Arrazat et al., 2023) |
| 116 | ML | N/A | 160 | Not reported | National | Quantitative | Correlation Analysis, Regression Analysis (MLP) | ✓ | (Parcesepe et al., 2023) |
| 117 | Sensory technologies, ML | N/A | 62 | 21 - 58 | Local | Quantitative | PCA, ANOVA, Regression Analysis (ANN), Classification Analysis (SVM) | N/A | (Gupta et al., 2022) |
| 118 | Transactional analytics | N/A | 795 | 18 - 24 | Local | Quantitative | Cluster Analysis | N/A | (Morris et al., 2020) |
| 119 | Personalized services | N/A | 207 | 18 - 30 | Local | Quantitative | SEM | N/A | (M. T. T. Nguyen et al., 2021) |
| 120 | Big Data, online reviews analytics, ML | N/A | 14454* | Not reported | National | Quantitative | Frequency Analysis, LDA, Sentiment Analysis (Bayesian Classifier) | N/A | (C. Zhang et al., 2021) |
| 121 | Transactional analytics | N/A | 515 | 32 - 73 | Local | Quantitative | Cluster Analysis | N/A | (Jiyoung & Heedae, 2020) |
| 122 | Transactional analytics | N/A | Not reported | 40 - 70+ | Local | Quantitative | Regression analysis | ✓ | (Green et al., 2020) |
| 123 | VR | N/A | 99 | Mean age: 30 | Local | Quantitative | ANOVA, t-test | Ν/A | (Blom et al., 2021) |
| 124 | Service-bots | ΤΑΜ | 514 | 50 - 70+ | National | Quantitative | CFA, SEM | N/A | (Lee et al., 2021) |
| 125 | Big data: transactional analytics | N/A | 299260 | Not reported | Local | Quantitative | Cluster Analysis | N/A | (Clark et al., 2021) |
| 126 | Big data: transactional analytics, ML | N/A | 150000 | Not reported | National | Quantitative | Regression Analysis (SVM), Correlation Analysis | ✓ | (Miliou et al., 2021) |
| 127 | VR | N/A | 114 | 18 - 41 | Local | Mixed | Thematic Analysis, Frequency Analysis, Sentiment Analysis, EFA, CFA, PLS-SEM, t-test, Mediation Analysis | N/A | (Park et al., 2021) |
| 128 | VR | N/A | 1452 | Mean age: 35 | National | Quantitative | ANOVA | N/A | (Blitstein et al., 2020) |
| 129 | VR | N/A | 318 | Mean age: 30 | National | Quantitative | Regression Analysis, ANOVA | N/A | (Hoenink et al., 2020) |
| 130 | Other | N/A | 125 | 21 - 66 | National | Quantitative | Regression Analysis | N/A | (Shin et al., 2020) |
| 131 | MR | N/A | 120 | 18 - 65 | Local | Quantitative | Regression Analysis, ANOVA | ✓ | (Low et al., 2021) |
| 132 | Apps with personalized services | Privacy Calculus | 797 | 18 - 75 | National | Quantitative | Correlation Analysis, CFA, SEM | N/A | (Reinders et al., 2020) |
| 133 | Apps with personalized services | N/A | 429 | 18 - 78 | National | Quantitative | Regression Analysis, Cluster Analysis | N/A | (Pérez-Troncoso et al., 2021) |
| 134 | Personalized services | Random Utility Theory | 303 | Not reported | National | Quantitative | Choice-based Conjoint Analysis, Classification Analysis | N/A | (Calegari et al., 2018) |
| 135 | Big Data, SMA | N/A | 205* | Not reported | Local | Qualitative | Content Analysis | N/A | (Ariyasriwatana & Quiroga, 2016) |
| 136 | Big Data: transactional analytics, ML | N/A | 22612* | Not reported | National | Quantitative | Correlation Analysis, ANOVA, Regression Analysis (Linear & MLP-based) | Ν/A | (Mu, 2019) |
| 137 | Big Data, ML | Consumption Value Theory | 829 | 16 - 35 | National | Quantitative | Frequency Analysis, Semantic Analysis (LSA), SEM | ✓ | (T. Y. Chen et al., 2016) |
| 138 | Big data: transactional analytics | Rational Choice Theory | 68934 | 18 - 60+ | Local | Quantitative | Regression Analysis, Classification Analysis | N/A | (Luo et al., 2015) |
| 139 | Big data, transactional analytics | N/A | 70447 | 55 - 60 | National | Quantitative | Regression Analysis | N/A | (Grummon & Taillie, 2017) |
| 140 | Big data, SMA | N/A | 26269* | Not reported | Not reported | Mixed | Correlation Analysis, Cluster Analysis, Frequency Analysis, Analysis of interviews (ISM) | ✓ | (Mishra et al., 2017) |
| 141 | VR | N/A | 947 | 18 - 65+ | National | Quantitative | Regression Analysis | N/A | (Payne Riches et al., 2019) |
| 142 | VR | N/A | 123 | Not reported | National | Quantitative | Regression Analysis | ✓ | (Waterlander et al., 2015) |
| 143 | Big data: transactional analytics | N/A | 598102 | 20 - 80 | Local | Quantitative | Correlation Analysis, Regression Analysis | N/A | (Tian et al., 2018) |
| 144 | Big data, ML, personalized services | N/A | 3000000* | Not reported | National | Quantitative | TF-IDF, Cluster Analysis, Classification Analysis (SVM, DT, RF) | N/A | (Kanavos et al., 2018) |
| 145 | VR, EEG | N/A | 30 | Not reported | Local | Quantitative | Correlation Analysis | N/A | (Biercewicz et al., 2024) |
| 146 | ML | N/A | 957 | 18 - 65+ | National | Quantitative | PCA, Cluster Analysis, Classification Analysis (RF, RT), ANOVA | ✓ | (Burke et al., 2025) |
| 147 | Big Data: Online reviews analytics | SOR model, TPB | 39.078* | Not reported | Global | Quantitative | Word Frequency Analysis, Sentiment Analysis (NLP-based), Time-series Analysis | N/A | (Demydyuk et al., 2024) |
| 148 | Big Data, SMA | N/A | Not reported | Not reported | National | Quantitative | Frequency & Sentiment Analysis (NLP-based), Time-series Analysis | N/A | (Fisk et al., 2024) |
| 149 | Video recording, ML | N/A | 26 | 18 - 24 | Local | Mixed | Thematic Analysis, Sentiment Analysis, LDA, Classification Analysis (CNN) | ✓ | (Gul et al., 2024) |
| 150 | ML | Theory of Diffusion of Innovation | 281 | Not reported | Local | Quantitative | Cluster Analysis, Classification Analysis (Bayesian classifier), SNA | Ν/A | (Huo et al., 2024) |
| 151 | SMA, ML | N/A | 8721* | Not reported | National | Quantitative | LDA, Frequency Analysis, Classification Analysis (RF, RT, SVM, CART, DT), Sentiment Analysis | N/A | (Ju, 2024) |
| 152 | VR | N/A | 63 | 19 - 59 | Local | Quantitative | ANOVA, Regression Analysis, Correlation Analysis, Factor Analysis | ✓ | (Man et al., 2024) |
| 153 | ML | N/A | 31 | 18 - 24 | Local | Quantitative | Time-series analysis (ESN), t-test | Ν/A | (Natsume & Okamoto, 2024) |
| 154 | Personalized services (AI) | Risk Aversion Theory, Risk Perception Theory, Rational Choice Theory | 613 | Mean age: 43 | National | Quantitative | ANOVA, Correlation Analysis, Mediation Analysis, Regression Analysis | N/A | (Rohden & Espartel, 2024) |
| 155 | Transactional analytics, ML | N/A | 51290* | Not reported | Global | Quantitative | Cluster Analysis (DBSCAN) | ✓ | (Sakaline & Buics, 2024) |
| 156 | ML | TPB, IBT, GFT | 334 | 23 - 45+ | Local | Quantitative | Classification Analysis (DT) | ✓ | (Sukumaran & Majhi, 2024b) |
| 157 | Online reviews analytics, ML | AISAS model | 23138* | Not reported | Local | Mixed | Sentiment Analysis (NLP-based), Classification Analysis (Bayesian classifier), fsQCA | ✓ | (Tan et al., 2025) |
| 158 | AI- and AR-driven nutrition app with personalized services | TAM | 12 | 22 - 54 | Local | Mixed | Frequency Analysis | N/A | (Campos et al., 2024) |
| 159 | VR | N/A | 60 | Not reported | Local | Mixed | Cluster Analysis, t-test | N/A | (Zulkarnain et al., 2024b) |

*refers to the number of evaluation records, mainly online reviews or transactional entries, and not necessarily to individual participants, as one participant may have contributed more than one records.
